# Multiscale Simulations Identify Origins of Differential Carbapenem Hydrolysis by the OXA-48 β-Lactamase

**DOI:** 10.1021/acscatal.1c05694

**Published:** 2022-04-04

**Authors:** Viivi H. A. Hirvonen, Tal Moshe Weizmann, Adrian J. Mulholland, James Spencer, Marc W. van der Kamp

**Affiliations:** † School of Biochemistry, University of Bristol, University Walk, Bristol BS8 1TD, U.K.; ‡ Centre for Computational Chemistry, School of Chemistry, University of Bristol, Cantock’s Close, Bristol BS8 1TS, U.K.; § School of Cellular and Molecular Medicine, University of Bristol, University Walk, Bristol BS8 1TD, U.K.

**Keywords:** carbapenem hydrolysis, OXA-48 β-lactamase, meropenem, imipenem, hydrogen bonding, QM/MM simulations

## Abstract

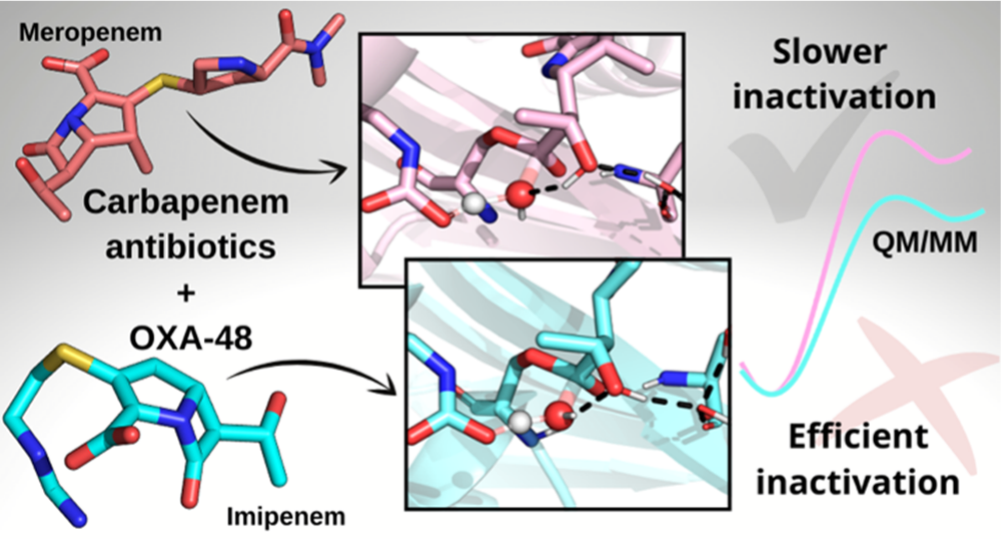

OXA-48 β-lactamases are frequently encountered in bacterial infections caused by carbapenem-resistant Gram-negative bacteria. Due to the importance of carbapenems in the treatment of healthcare-associated infections and the increasingly wide dissemination of OXA-48-like enzymes on plasmids, these β-lactamases are of high clinical significance. Notably, OXA-48 hydrolyzes imipenem more efficiently than other commonly used carbapenems, such as meropenem. Here, we use extensive multiscale simulations of imipenem and meropenem hydrolysis by OXA-48 to dissect the dynamics and to explore differences in the reactivity of the possible conformational substates of the respective acylenzymes. Quantum mechanics/molecular mechanics (QM/MM) simulations of the deacylation reaction for both substrates demonstrate that deacylation is favored when the 6α-hydroxyethyl group is able to hydrogen bond to the water molecule responsible for deacylation but disfavored by the increasing hydration of either oxygen of the carboxylated Lys73 general base. Differences in free energy barriers calculated from the QM/MM simulations correlate well with the experimentally observed differences in hydrolytic efficiency between meropenem and imipenem. We conclude that the impaired breakdown of meropenem, compared to imipenem, which arises from a subtle change in the hydrogen bonding pattern between the deacylating water molecule and the antibiotic, is most likely induced by the meropenem 1β-methyl group. In addition to increased insights into carbapenem breakdown by OXA β-lactamases, which may aid in future efforts to design antibiotics or inhibitors, our approach exemplifies the combined use of atomistic simulations in determining the possible different enzyme–substrate substates and their influence on enzyme reaction kinetics.

## Introduction

The World Health Organization describes antibiotic resistance as “...one of the biggest threats to global health, food security, and development today”.^2^ Antibiotic resistance arises naturally and evolved long ago,^3^ but its emergence and dissemination have been considerably accelerated by the current excessive use of antibacterial drugs.^4,5^ This evolving resistance not only complicates standard medical practices but also has additional expensive implications, for example, for the global economy and food production.^6−8^ Moreover, we are currently living in the so-called antibiotic discovery void^9^ where discovering new and safe antibacterials, especially for Gram-negative bacteria, is difficult, time-consuming, and often unprofitable for big pharmaceutical companies.^10,11^ β-Lactam antibiotics offer broad-spectrum antibacterial activity against Gram-negative bacteria and remain the most prescribed drugs in clinical practice.^12^ The importance of β-lactams in healthcare has been highlighted by the World Health Organization, which includes multiple different β-lactam antibiotics in their Model List of Essential Medicine.^13^ All of these antibiotics contain a four-membered β-lactam ring, which ensures antibiotic binding to penicillin-binding proteins and, consequently, inhibition of bacterial cell wall biosynthesis.^14,15^ Clinically used β-lactam compounds can be divided into four different groups: penicillins, cephalosporins, carbapenems, and monobactams, of which carbapenems play a critical role as potent antibiotics reserved for the most serious Gram-negative infections where alternatives are limited.^16^

Emerging resistance against β-lactams is evident, and especially in Gram-negative bacteria, β-lactamase enzymes are the main resistance mechanism against these drugs.^17^ β-Lactamases block antibiotic action by hydrolyzing the β-lactam ring, which impairs efficient antibiotic binding to their ultimate target in cells. The Ambler sequence-based classification divides β-lactamases into four major subgroups: serine-β-lactamases (SBLs) comprising classes A, C, and D and metallo-β-lactamases (MBLs), class B.^18^ The hydrolysis mechanism differs between SBLs and MBLs as SBLs utilize a nucleophilic serine residue and MBLs employ zinc cofactors.^17^ Class D SBLs are referred to as OXA (oxacillinase) enzymes, stemming from their activity against the isoxazolyl penicillin oxacillin,^19^ and they are currently of interest due to their wide distribution and the ability of many members of the group to inactivate carbapenems. The OXA enzymes include five subgroups of recognized carbapenemases: the OXA-23, OXA24/40, OXA-51, and OXA-58 β-lactamases are mainly found in *Acinetobacter baumannii*, while OXA-48-like β-lactamases are mostly encountered in Enterobacterales.^20^

**Figure 1:**
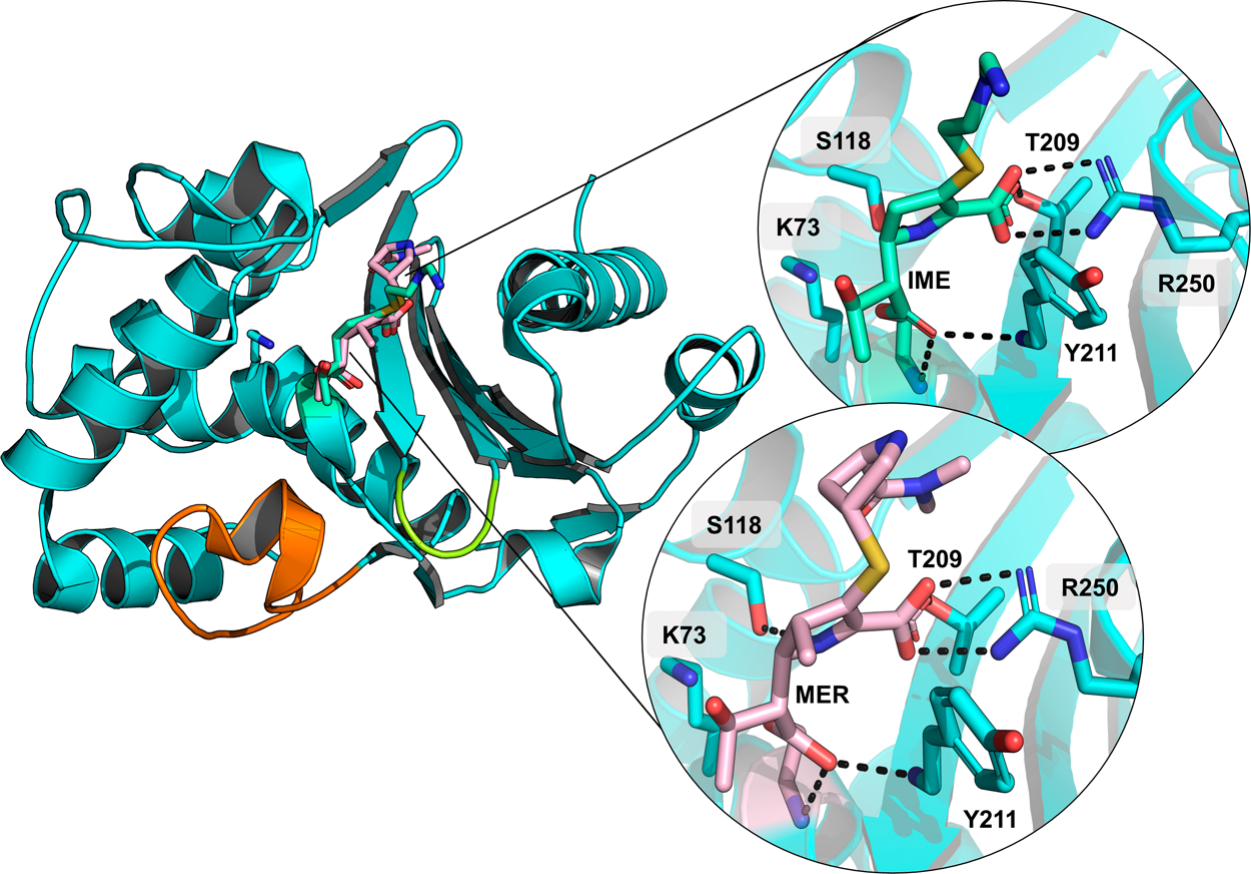
Crystal structures of OXA-48 complexed with carbapenems. Acyl-enzyme (AC) structures of OXA-48 with IME (PDB ID 6P97, green sticks) and MER (PDB ID 6P98, light pink sticks) show a highly similar binding pose for both substrates, where main differences lie in the orientation of the carbapenem C2 “tail” group.^1^ The Ω-loop is highlighted in orange, the β5−β6-loop in yellow, and relevant active site interactions in dashed black lines. The carbapenem pyrroline ring is modeled as the Δ2-tautomer in both structures.

In Enterobacterales, OXA-48 β-lactamases are among the most commonly present carbapenemases in clinical samples.^21^ Their activity is relatively specific toward imipenem (IME), but other carbapenem substrates [such as meropenem (MER) and ertapenem] are also hydrolyzed, albeit slowly.^22^ The specific origin of this imipenemase activity is not well established, even though variations in measured hydrolysis rates between point variants of OXA-48 hint at structural moieties contributing to specific hydrolytic phenotypes (Figure 1). In OXA-163, a partial deletion of the β5−β6 loop (Arg214–Pro217) and one amino acid substitution (Ser212Asp) expand the hydrolysis profile to accommodate expanded-spectrum oxyimino cephalosporins (such as ceftazidime) at the expense of efficient IME breakdown.^23^ Further studies show that the β5−β6 loop plays a role in the acquired carbapenemase activity. The engineering the OXA-48 β5−β6 loop into the non-carbapenemase OXA-10 enhances its carbapenemase activity.^24^ Conversely, replacing the β5−β6 loop in OXA-48 with that of OXA-18 also alters the measured carbapenemase activity (lower *k*_cat_ values).^25^ Site-directed mutagenesis studies of OXA-48 variants indicate that residue 214 (arginine in the wild-type OXA-48) is essential for efficient carbapenem hydrolysis.^26^ In recent years, structural studies have yielded a variety of crystal structures of OXA-48 in complex with carbapenems, which shed new light on the acylenzyme (AC) intermediate state.^1,27−30^ Intriguingly, although the β5−β6 loop is suggested to influence carbapenem activity, the only interaction observed between the substrate and residues within this loop (Thr213–Lys218) is a water-mediated contact between IME 6α-hydroxyethyl hydroxyl and Thr213.^1,30^ Furthermore, bound carbapenem tail groups (C2 substituents) appear to be dynamic and are able to adopt multiple conformations, which suggest that they do not form strong, specific interactions with the enzyme active site.^29^

**Scheme 1:**
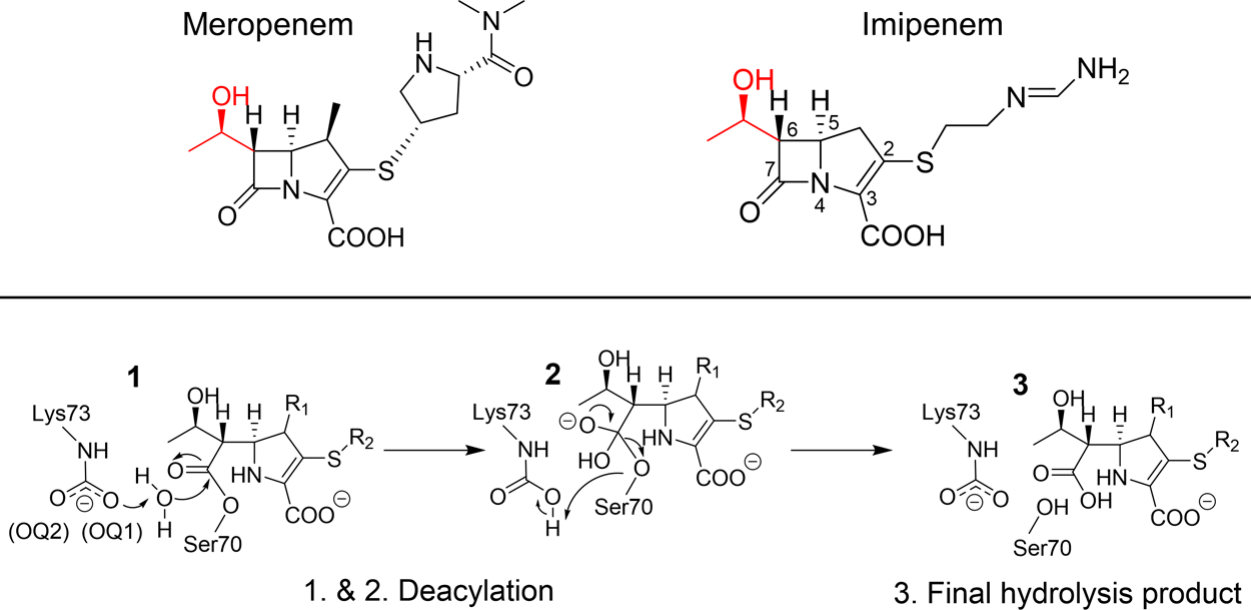
Top: Structures of MER and IME with the 6α-Hydroxyethyl Group Is Highlighted in Red; Bottom: Deacylation Mechanism in OXA-48 with a Carbapenem Substrate (Δ2 Tautomer); Starting from the AC, the Antibiotic Is Deacylated via TI Formation (1 → 2), Which Collapses to Yield the Hydrolyzed Antibiotic (3)

The generalized β-lactam hydrolysis mechanism for SBLs consists of acylation, followed by deacylation (Scheme 1).^17^ Both acylation and deacylation reactions include the formation of a short-lived tetrahedral intermediate (TI) through a nucleophilic attack; the respective TI species collapses to yield either a covalent AC structure (after acylation) or the final hydrolyzed product (after deacylation). In both reactions, the nucleophile [conserved serine (Ser70) in acylation and a water molecule (deacylating water, DW) in deacylation] is activated via proton abstraction by a general base. For OXA enzymes, this general base is a carboxylated lysine residue (Lys73).^31,32^ Notably, Lys73 needs to be carboxylated for optimal activity; this carboxylation is reversible and pH-dependent; that is, more carboxylation is observed at higher pH values.^31^ At lower pH values, protonation of Lys73:Nζ would lead to decarboxylation.^33^ Based on pH dependence studies of the reaction between OXA-10 and penicillin or nitrocefin, the p*K*_a_ of the carboxylated Lys73 is expected to be ∼5.8–6.2.^31^ For carbapenems, the pyrroline ring can undergo Δ2 → Δ1 tautomerization in the AC state; the Δ1 tautomer also has two stereoisomers (*R* and *S*). For class A SBLs, the Δ2 tautomer has been suggested to be the catalytically competent form, whereas the Δ1 form would essentially inhibit the enzyme.^34^ For OXA-48 enzymes, all three tautomers have been observed in AC crystal structures,^1,28−30^ but, based on NMR studies, the hydrolysis product is suggested to be either the Δ2 or *R*-Δ1 tautomer.^35^

Kinetic measurements suggest that for OXA-48-like β-lactamases, deacylation is the rate-limiting step in carbapenem breakdown.^30^ These authors suggested that the impaired imipenemase activity in the ESBL-like OXA-163, compared to OXA-48, is due to a larger active site, which would not constrain the substrate in deacylation-compatible conformations. Molecular dynamics (MD) simulations of the noncovalent complexes of OXA-48 and OXA-163 with MER and IME suggested some differences in mobility between the substrates. However, the measured *K*_M_ values for OXA-48 with IME and MER are very similar (according to one assay, 11 and 13 μM, respectively),^22^ which indicates that there is unlikely to be any significant difference in the stabilities of the respective Michaelis complexes. The difference in the inactivation efficiency of IME compared to MER is thus primarily related to differences in the rate of the deacylation step, and it is therefore essential to consider this reaction when seeking to understand and explain activity differences. To analyze differences in activity for carbapenems in atomistic detail, we here simulate TI formation in deacylation, that is, the expected rate-limiting step, of both IME and MER by OXA-48 using combined quantum mechanics/molecular mechanics (QM/MM) simulations. Our simulations support the hypothesis that the AC state arising from carbapenem acylation is dynamic in nature. Furthermore, we identify conformations of the 6α-hydroxyethyl group that allow for efficient deacylation. Additionally, active site hydration around the carboxylated Lys73 is observed to affect the calculated free energy barriers for deacylation, as we previously observed for hydrolysis of the expanded-spectrum oxyimino cephalosporin ceftazidime by OXA-48 enzymes.^36^ Analysis of the reaction simulations shows that efficient carbapenem breakdown results both from a decrease in hydration around carboxy-Lys73 and from subtle changes in hydrogen bonding between the substrate and the catalytic water molecule. These results provide detailed insight into the causes of differences in enzyme activity against different antibiotics, information potentially useful in understanding and combating antimicrobial resistance.

## Methods

Computational methods and details of the system setup are described in detail in the Supporting Information. To summarize, models of OXA-48 with IME and MER were prepared based on the corresponding AC crystal structures (PDB IDs 6P97(1) and 6P98(1) for IME and MER, respectively). The ff14SB parameter set was used for the protein;^37^ parameters and partial charges for nonstandard residues (acylated carbapenems and carboxylated lysine) were derived with the R.E.D. Server.^38^ Both systems were energy-minimized and heated from 50 to 300 K (in 20 ps), and their dynamics in the AC state were simulated for 200 ns using Langevin dynamics (collision frequency, 0.2 ps^–1^) with a 2 fs timestep. Five independent simulations for each AC system were run. All bonds involving hydrogens were restrained using the SHAKE algorithm. Starting structures for QM/MM^39^ modeling were chosen from MD simulations based on visual inspection of the active site hydration pattern and the 6α-hydroxyethyl orientation; this orientation was kept from changing during subsequent QM/MM umbrella sampling (US) MD by applying a weak dihedral restraint (except in the case of orientation I). Free energy barriers for the first (rate-limiting) step of deacylation for the different active site conformations were determined from three separate QM/MM US calculations for each conformation.^40^ Two reaction coordinates were employed in US: one for the nucleophilic attack and the other for the proton transfer, as in previous simulations of deacylation in SLBs.^36,41−43^ The sampling time in each window was 2 ps, and DFTB2 (SCC-DFTB)^44−46^ was used as the QM method for regions consisting of 43 and 46 atoms (including link atoms) for IME and MER, respectively (Figure S1). Free energy surfaces (FESs) were constructed from 399 individual US windows. The weighted histogram analysis method (WHAM)^47,48^ was used to construct the FESs, and the minimum energy paths were analyzed using the minimum energy path surface analysis (MEPSA) program.^49^ All simulations and trajectory analyses were done using the Amber18 software package^50^ (pmemd.cuda^51−53^ for MM MD and SANDER for QM/MM calculations).

## Results and Discussion

### Conformational Dynamics of Carbapenem:OXA-48 ACs

AC dynamics for both IME and MER complexed with OXA-48, each in the Δ2 (enamine) configuration, were explored by running five 200 ns MM MD simulations for each complex. The first 50 ns were excluded from trajectory analysis to allow time for equilibration. For both carbapenems, the salt bridge between the C3 carboxylate and Arg250 was preserved during simulations, and the C7 carbonyl stayed in the oxyanion hole formed by the backbone amides of Ser70 (nucleophile) and Tyr211. The carbapenem C2 (tail) substituents sampled a range of conformations during the simulations, consistent with previous suggestions based on structural analysis.^29^ Clustering the substrate poses based on their heavy-atom RMSD yielded four distinct clusters per substrate, which differ by 0.8–1.8 and 1.7–2.5 Å for IME and MER, respectively, from the poses in the corresponding crystal structures (Figure S2, Table S1 and the Supporting Information section Acylenzyme Clustering). The main deviations between cluster centroids and the crystal structure coordinates are due to the positions of the C2 tail groups as the pyrroline ring and its substituents are anchored in place by hydrogen bonds to the oxyanion hole and the salt bridge with Arg250. For the crystal structures 6P97 and 6P98, there is only limited electron density beyond the sulfur atom for both IME and MER, so the deposited coordinates may not completely reliably depict the actual substrate binding poses. Additional clustering on the active site residues (explained in further detail in the Supporting Information) implies that there may be slight differences also in the positions of active site residues Lys73 and Tyr157 as well as those of the substrate (Figure S3 and Table S2).

**Figure 2:**
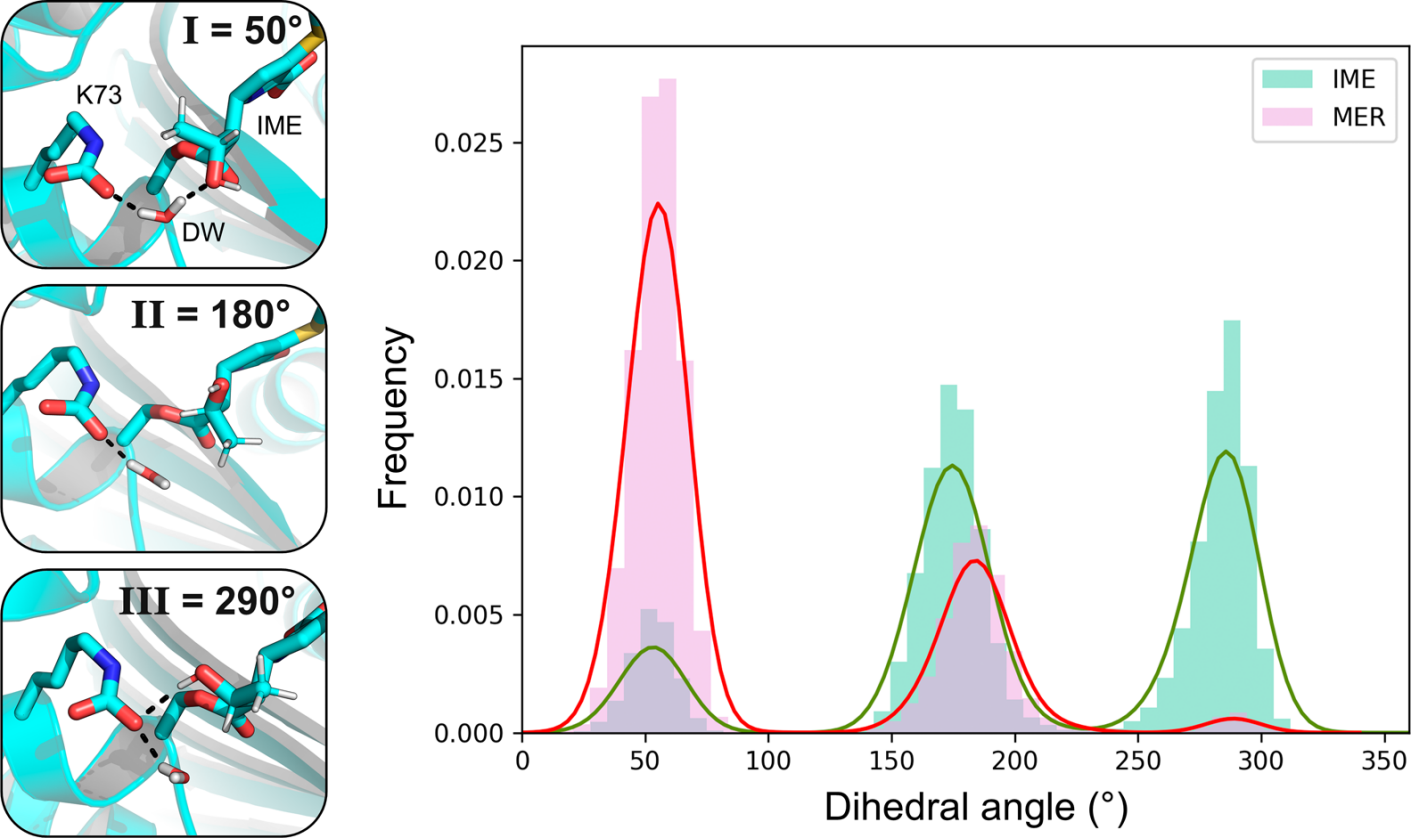
Conformational behavior of the carbapenem 6α-hydroxyethyl group. Left: 6α-Hydroxyethyl group can assume three different orientations, which can be distinguished by the C7–C6–C–O dihedral angle values. When the dihedral is around 50° (orientation I), the hydroxyl group is hydrogen bonded with the DW, and in the 180° orientation (II), the hydroxyl group can only interact with the solvent. In the 290° orientation (III), the hydroxyl group donates a hydrogen bond to the carboxylated Lys73. Right: Distribution of sampled dihedral values during MM MD simulations of the IME and MER ACs (5 × 150 ns per carbapenem).

During MM MD, the carbapenem 6α-hydroxyethyl group was able to rotate to occupy three different orientations, which can be distinguished by the value of the C7–C6–C–O dihedral angle: around 50, 180, or 290°, henceforth referred to as orientations I, II, and III, respectively (Figure 2). The 6α-hydroxyethyl orientation affects interactions in the active site because its hydroxyl group can hydrogen bond either with the DW (I) or with the Lys73 carboxylate (III) or stay close to the crystallographically observed pose, in which its methyl group is positioned next to the DW and points toward Leu158 (II, Figure 2). The starting orientation of 6α-hydroxyethyl for both carbapenems is II, as in the crystal structures used in model construction. During MD simulations, this side chain is free to move and sample all three orientations. For MER, orientation I is sampled more than II, while III is sampled only minimally (Figure 2). Conversely, both orientations II and III are sampled more than I for IME. The free energy difference between the different orientations of the 6α-hydroxyethyl group was estimated by calculating the ratio of MD trajectory frames corresponding to each orientation (*Z*) and using Δ*G* = *RT*ln(*Z*), where *R* is the molar gas constant and *T* the simulation temperature (300 K). For IME, the lowest free energy state is orientation II, with slightly higher relative energies of 0.6 and 0.2 kcal/mol for orientations I and III, respectively. For MER, orientation I has the lowest free energy, orientation II is slightly higher (0.6 kcal/mol), but orientation III is significantly higher (2.2 kcal/mol). The presence of a methyl group in the 1β position in MER (instead of a 1β proton in IME) may explain the relatively higher penalty for orientation III, as in this orientation, the 1β-substituent is located directly next to the 6α-hydroxyethyl moiety.

Previously, our QM/MM simulations indicated that Leu158 may play an important role in modulating active site hydration in the deacylation of ceftazidime by OXA-48-like enzymes.^36^ The orientation of Leu158 also differed initially between the two OXA-48/carbapenem systems as the Cβ–Cγ bond was rotated by 180° in the MER structure. To study if Leu158 has a similar effect on carbapenem hydrolysis to that observed for ceftazidime, its rotamers were first investigated by measuring the χ_1_ dihedral (N–Cα–Cβ–Cγ) in MM MD simulations. The distribution of sampled rotamers is presented in Figure S4. After the heating phase, Leu158 essentially always rotates away from the crystallographic *g*– orientation (χ_1_ ≈ 290°) to the *t* orientation (χ_1_ ≈ 180°) to allow space for the 6α-hydroxyethyl moiety, which in turn also permits for two water molecules to form hydrogen bonds with Lys73:OQ1. As the cephalosporin scaffold lacks a functional group similar to the 6α-hydroxyethyl group of carbapenems, typically bearing larger substituents in the β orientation at the equivalent 7-position, it is likely that Leu158 does not possess a similar role in carbapenem hydrolysis to that suggested for cephalosporins.

### Deacylation Efficiencies for Different Orientations of the 6α-Hydroxyethyl Group

Because the interactions of the 6α-hydroxyethyl group in the active site have been suggested to play a role in modulating β-lactamase activity toward carbapenems,^32^ deacylation free energy barriers were calculated separately for all three orientations of both IME and MER ACs observed in MD simulations. Starting structures for US were chosen from the 200 ns MM MD simulations following two criteria: that a potential DW was at a suitable distance for the nucleophilic attack and the 6α-hydroxyethyl orientation was that desired. For orientations II and III, the side-chain dihedral was restrained close to the reference values to avoid the substrate changing between orientations during the reaction (no restraints were needed for I as no side-chain rotation was observed during US). Overall barriers for deacylation were determined by combining sampling from three separate US calculations for each AC conformation (with different starting structures), with standard deviations calculated between the free energy barriers for individual US simulations (Table S3). More details of the US setup and analysis are available in the Supporting Information.

**Figure 3:**
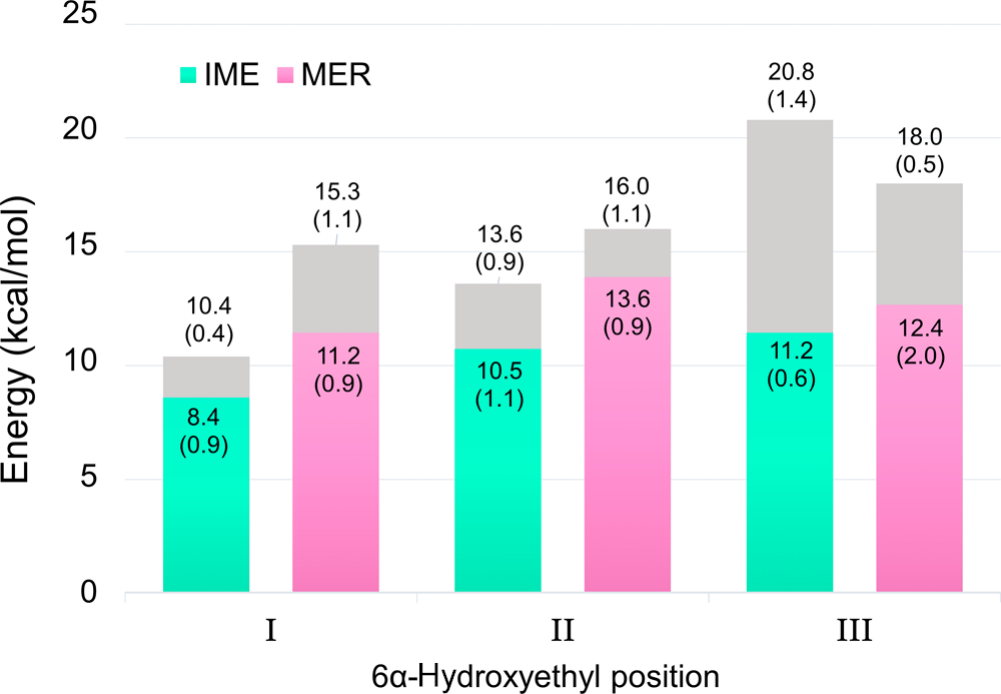
Free energy barriers for deacylation of carbapenem ACs with the 6α-hydroxyethyl group in the three different orientations. Each bar includes the barrier obtained with a single water molecule hydrogen bonded to Lys73:OQ2 (lowest barrier, in color; see Figure 4 for depiction of OQ2) and the barrier obtained with two water molecules hydrogen bonded to Lys73:OQ2 (highest barrier, in gray). Each barrier is derived from three individual US simulations, with standard deviations in parenthesis. IME: green, MER: pink.

**Figure 4:**
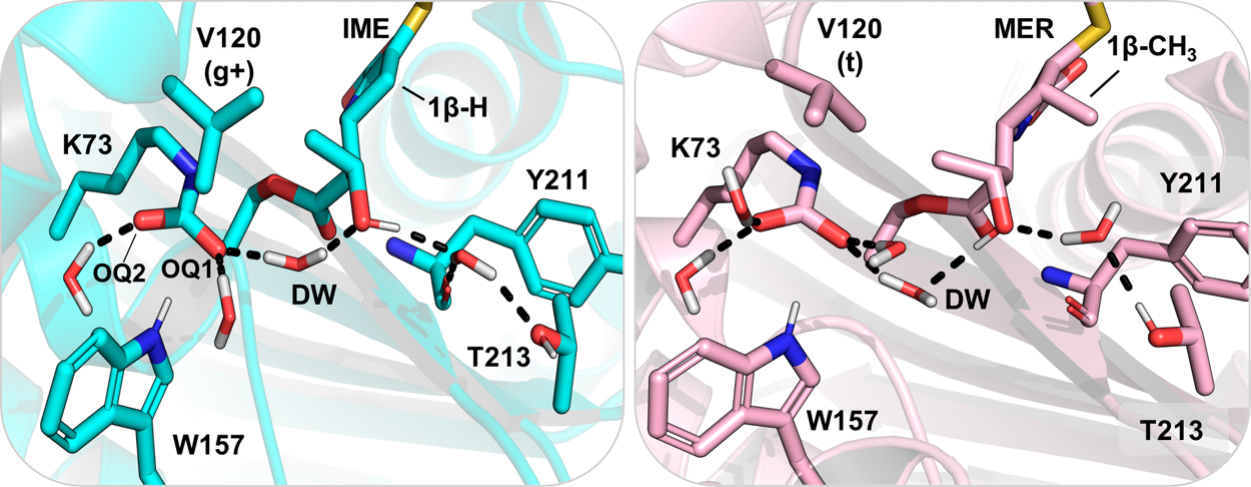
Alternative hydrogen bond configurations found with 6α-hydroxyethyl in orientation I. Left: Active site of OXA-48 with IME in hydrogen bond configuration (1). Val120 adopts the g+ rotamer, and consequently, only one water molecule forms a hydrogen bond with Lys73:OQ2. 6α-Hydroxyethyl is in orientation I and donates a hydrogen bond to a water lodged between the Tyr211 backbone and Thr213. Right: Active site interactions of OXA-48 with MER in hydrogen bond configuration (2). Val120 is in its *t* rotameric state, which allows for two waters to hydrogen bond with both Lys73 carboxylate oxygens. 6α-Hydroxyethyl is in orientation I but donates a hydrogen bond to the DW.

Calculated deacylation free energy barriers for the ACs formed by IME and MER with 6α-hydroxyethyl in each of the three orientations are shown in Figure 3. For all orientations, two barriers are shown, corresponding to the two different hydration states around the general base. The lower barrier (in color) corresponds to a state with only one water molecule hydrogen bonded to Lys73:OQ2 and one or two water molecules hydrogen bonded to Lys73:OQ1, while the higher barrier corresponds to a state with two water molecules hydrogen bonded to both carboxylate oxygens (Figure 4, carboxylate oxygens labeled in Scheme 1). For all hydration states, the calculated barriers follow the same trend of I < II < III; that is, the lowest barriers are calculated for orientation I. Notably, the barriers are consistently underestimated due to the QM method used (DFTB2), as is generally found for this method for similar reactions.^42,43^ This underestimation likely also causes an underestimation of the stability of the TI compared to that of the transition state (TS; see, e.g., the small molecule benchmark calculations the Supporting Information section “Benchmarking”), but TI minima were still located in our FESs (likely due to stabilization by the enzyme environment). As the overall shape of the QM/MM potential energy surface (PES) is consistent when using DFTB2 or M06-2X/def2-TZVP as the QM method, it is reasonable to expect that the underestimation of TI stability with DFTB2 does not affect the trends in reaction barriers (Supporting Information section “Benchmarking”). Taking into account an underestimation of ∼8 kcal/mol, as indicated by comparison of DFTB2 to SCS-MP2/aug-cc-pVTZ (Supporting Information section “Benchmarking”), the lowest barriers are in good agreement with the experiment (see further the section “Comparison with Experimental Data”). Importantly, we expect our protocol for obtaining free energy barriers using semiempirical QM methods to be a reliable indicator of relative energetic trends between different enzyme active site conformations; we have demonstrated this previously in the studies of deacylation of β-lactam ACs for both class A (with MER) and D SBLs.^36,43^

As discussed above and in ref (36), increased hydration around the proton-accepting Lys73:OQ1 impairs deacylation in ceftazidime hydrolysis. A similar phenomenon was observed for carbapenems, with the additional observation that hydration around the second carboxylate oxygen (Lys73:OQ2) also affects reactivity. In orientation I, the average number of hydrogen bonds Lys73:OQ1 accepts during the reaction is 2.4 (±0.1 standard deviation, calculated from the US minimum free energy path trajectories), which aligns with OQ1 being hydrogen bonded to two water molecules and partly to Trp157. The two subpopulations with different deacylation barriers arise from a change in hydration around Lys73:OQ2. For the lower barriers in Figure 3, the number of hydrogen bonds to OQ2 is 1.3 (±0.1) and for the higher barriers 2.2 (±0.1) for orientation I. The lowest calculated deacylation barrier, 8.4 kcal/mol, is for IME in orientation I with one water molecule hydrogen bonded to OQ2 and two to OQ1 (Figure 4). The barrier increases by 2.0 kcal/mol when another solvent molecule donates a hydrogen bond to OQ2. For MER, the barrier is raised by 4.1 kcal/mol upon introduction of an additional water molecule close to OQ2. The hydration effect around Lys73:OQ2 indicated here has an apparently smaller effect on the calculated barriers than that of hydration around Lys73:OQ1 since the presence of an additional water molecule hydrogen bonded to OQ1 raised the barrier for ceftazidime deacylation by approximately 5 kcal/mol.^36^

Orientation II (corresponding to a dihedral angle of between 147 and 192° depending on the structure and the protein chain) is observed in most OXA-48:carbapenem AC crystal structures. In this orientation, no part of the 6α-hydroxyethyl moiety interacts with either the DW or with Lys73, so the antibiotic may possibly not interfere with the reactive atoms. However, calculated deacylation barriers are increased by 2.1 kcal/mol for IME and by 2.4 kcal/mol for MER when comparing orientation II against I (in which only one water molecule is hydrogen bonded to OQ2). Having two water molecules donating hydrogen bonds to both OQ1 and OQ2 further raises the calculated barriers to 13.6 and 16.0 kcal/mol for IME and MER, respectively. Therefore, our simulations suggest that II is not the most deacylation-competent AC orientation. Additionally, orientation II might hinder the positioning of the DW in the active site in proximity to the electrophilic acyl carbon. For 93 and 87% of the simulation times for the IME and MER ACs in orientation II, respectively, the distance between the AC electrophilic carbon and the closest water molecule falls beyond 4 Å (an arbitrary threshold distance for a feasible nucleophilic attack; Figure S5). This is likely due to the 6α-hydroxyethyl methyl group partly occupying the space in the binding pocket for the deacylating water molecule and thereby forcing this water further away from the AC. This is reflected in deposited crystal structures as a DW candidate that is suitably positioned for the nucleophilic attack is not observed in any OXA-48/carbapenem complex.^1,27−30^ In a previous study (mainly based on MD), orientation II was observed to obstruct the positioning of the DW in the active site.^32^ Docquier et al. concluded that only a slight repositioning of the methyl group of the 6α-hydroxyethyl side chain is needed to better accommodate a water molecule at a suitable distance for the nucleophilic attack. However, these conclusions are based on a single 10 ns MD simulation, which likely gives insufficient time to sample all available substrate orientations. Based on our MM MD simulations, as well as the calculated free energy barriers, orientation II is less likely to contribute to efficient deacylation of the carbapenem ACs. This is due to both an increase in energy required for deacylation and a lack of sampling of active site configurations that would be suitable for the AC carbonyl to undergo nucleophilic attack by an incoming water molecule.

The largest increase in energetics between the two hydration states is calculated for orientation III, where the barriers increase by 9.6 and 5.6 kcal/mol for IME and MER, respectively, when the hydration state is changed. For the lower barriers, OQ1 and OQ2 form on average 2.0 (±0.1) and 1.4 (±0.1) hydrogen bonds, respectively, for the IME and MER complexes, while for the higher barriers, the equivalent numbers are 2.8 (±0.1) and 2.1 (±0.2, data not shown). For the lower barriers, Leu158 has not (yet) rotated from the *g*– to the *t* rotamer (Figure S4) as the starting structures were chosen almost directly after the heating phase. The *g*– rotamer of Leu158 allows space only for the DW positioned near Lys73:OQ1, which was inserted into the active site in the starting model. Furthermore, only one water molecule donates a hydrogen bond to OQ2. Upon MD equilibration, Leu158 rotates, allowing for active site hydration to change to two water molecules hydrogen bonding to both carboxylate oxygens each. Subsequently, only the “high barrier” hydration state is sampled. This explains the large increase in activation free energy when comparing the two hydration substates for orientation III, as two water molecules are located near Lys73, as opposed to only one water molecule close to Lys73:OQ2 (as for orientations I and II). Therefore, our simulations indicate that III is the AC orientation that is the least competent for deacylation for the equilibrated system (in which Leu158 has rotated). Experimentally, this AC orientation is seen in the crystal structure of OXA-48 with hydrolyzed, noncovalently bound IME (PDB ID 6PK0),^28^ where the hydroxyethyl hydroxyl donates a hydrogen bond to the newly formed carboxylate group. In our MM MD simulations of the AC, the exchange between 6α-hydroxyethyl dihedral orientations is frequent (indicating a low energy barrier). This is probably also true for the hydrolyzed antibiotic, suggesting that rotation of this moiety can occur postdeacylation.

Further analysis of the US trajectories reveals that hydration around Lys73:OQ2 correlates with the rotamer of Val120. Valine has three rotamers for the χ_1_ dihedral (N–Cα–Cβ–Cγ1): the *g*+ rotamer around 50°, *t* around 180°, and *g*– around 300° (Figures 4 and S6). In the starting structures for simulations, Val120 is in the *t* orientation for both carbapenems (for MER, partial occupancy for both *t* and *g*– rotamers was observed in the deposited structure, but only the *t* rotamer was used in the computational model building).^1^ The rotameric state can switch to either *g*+ or *g*– during MD simulations (Figure S6). For the *g*+ rotamer, one of the methyl groups points directly toward Lys73, which only leaves space for a single water molecule next to Lys73:OQ2; this water is positioned to accept a hydrogen bond from Gln124 and to donate one to Lys73. Conversely, the *t* rotamer allows for a second water molecule to occupy the space between Lys73 and Val120, and this water molecule is able to donate hydrogen bonds to both Lys73:OQ2 and the Val120 backbone carbonyl. Val120 is part of motif II, which is formed by residues Ser118–Val120 and is conserved across class D β-lactamases.^32^ Together with Leu158, it forms the so-called “deacylating water channel” in the vicinity of Lys73; this hydrophobic patch partly shields the active site from bulk solvent.^1^ For other OXA enzymes, a similar water channel has been proposed to open upon substrate binding to allow for water ingress into the active site and therefore for efficient deacylation.^54,55^ For OXA-48, previous comparison of apoenzyme and AC structures shows that substrate binding shifts Val120 and Leu158 only slightly and that the water channel is more open than, for example, in OXA-23.^1^ Access of water into the catalytic position next to the substrate and Lys73 is necessary for antibiotic hydrolysis, but as we indicate above, any additional solvent in the active site will impair reactivity. In OXA-48, it appears that Val120 (and the specific rotamers that it samples) is an important gateway residue controlling approach of the bulk solvent to Lys73:OQ2. Our previous work (on ceftazidime hydrolysis in OXA-48-like enzymes) indicates that Leu158 modulates hydration around Lys73:OQ1.^36^ Notably, Val120 is mutated to a leucine in OXA-519, a single point mutant of OXA-48; this mutation results in an increase in measured hydrolysis for some 1β-methyl carbapenems, such as MER and ertapenem, but decreased imipenemase activity. Compared to OXA-48, OXA-519 also increases the proportion of β-lactone reaction products with respect to conventionally formed ring-opened hydrolysis products of MER.^56^ Furthermore, the Val120Leu mutation increases both *k*_cat_ and *K*_M_ for MER, indicating opposite effects on binding and hydrolysis.^57^ The exact effect of the Val120Leu mutation on carbapenem hydrolysis on the molecular level is therefore complex and remains to be determined.

### Comparison of Carbapenem Deacylation in Orientation I

As presented above, orientation I of the 6α-hydroxyethyl moiety is calculated to give the overall lowest deacylation free energy barriers for both carbapenems. The combined free energy surfaces (FESs) for the hydration state with lower free energy barriers are presented in Figure S7 for all three substrate orientations. In this section, we focus further on orientation I and the “reactive” active site configuration in which only one water molecule is hydrogen bonded to Lys73:OQ1 and two to Lys73:OQ2 (unless otherwise stated). For this AC conformation, two different hydrogen bonding arrangements in the active site are possible: the DW can donate a hydrogen bond to the 6α-hydroxyethyl hydroxyl group (named configuration 1), or the hydroxyl group can donate a hydrogen bond to the DW (configuration 2), as seen in Figure 4. In MM MD, configuration (1) is sampled for 87 and 86% of the simulation time for IME and MER, respectively. In addition to donating a hydrogen bond to the DW as in (2), the 6α-hydroxyethyl hydroxyl group can also donate a hydrogen bond directly to Lys73:OQ1 if the DW is displaced. This orientation of the carbapenem 6α-hydroxyethyl group may be the relevant one for β-lactone formation, which has been characterized as a side product for OXA-48-catalyzed carbapenem turnover, particularly of 1β-methyl carbapenems (such as MER).^56,58^ The β-lactone product has been proposed to form via intramolecular cyclization, where the hydroxyl group acts as a nucleophile and donates a proton to Lys73. If the reaction occurs without a bridging water molecule, that is, by a direct proton transfer between −OH and Lys73, lactonization is most likely lower in energy in orientation I than in III based on the trends observed for deacylation energetics.

**Figure 5:**
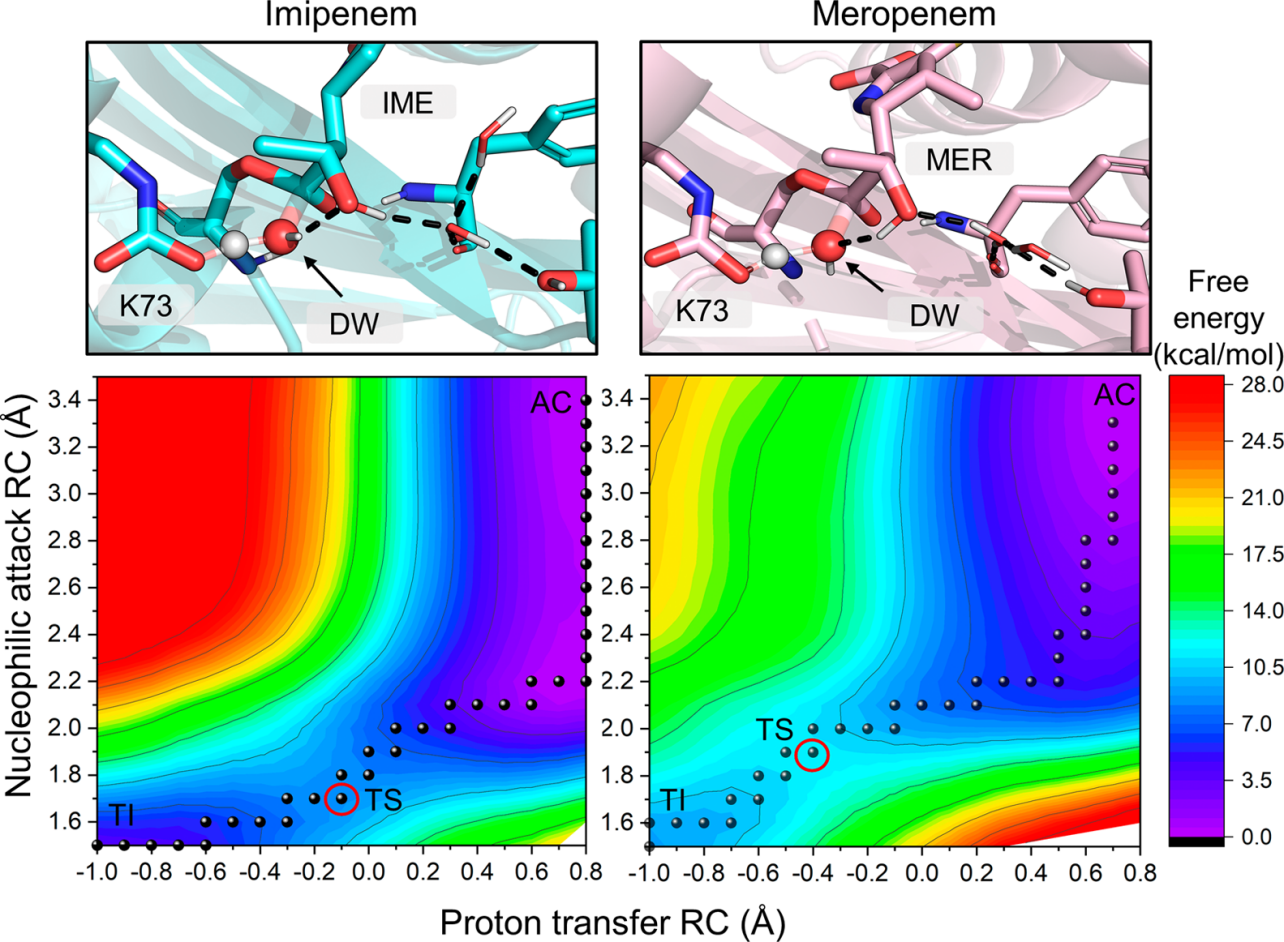
Free energy surfaces (FESs) and TS structures for alternative active site hydrogen bond configurations. Left: FES for IME deacylation for the lowest calculated barrier in orientation I (configuration 1). The DW donates a hydrogen bond to the carbapenem hydroxyl group. Right: FES for MER deacylation with the lowest calculated barrier in orientation I (configuration 2). The carbapenem hydroxyl group donates a hydrogen bond to the DW. AC = acylenzyme, TS = transition state (marked by a red circle), TI = tetrahedral intermediate.

For IME deacylation, both configurations (1) and (2) were observed in US. The lowest free energy barrier of 8.4 kcal/mol was calculated for configuration (1), and the barrier was increased by 2.0 kcal/mol for configuration (2). In addition to raising the free energy barriers, changing from (1) to (2) shifts the location of the TS on the FES. For (1), the TS is located approximately at values −0.1 and 1.7 Å for the proton transfer and nucleophilic attack reaction coordinates, respectively (Figure 5, left). However, for (2), the TS location on the FES shifts to around −0.5 and 2.0 Å (Figure S8), respectively. With active site configuration (2), the proton transfer has progressed further at the TS, whereas the approach of the DW oxygen to the acyl carbon is less advanced. This is potentially due to the additional hydrogen bond from the 6α-hydroxyethyl hydroxyl moiety decreasing the nucleophilicity of the DW, requiring the proton transfer reaction to have progressed further from the starting structure in the TS. Notably, a similar shift in the TS position on the FES is also observed in orientation III, where a water molecule donates a hydrogen bond to the DW instead of the 6α-hydroxyethyl group (Figure S7). Mulliken charge analysis of the key QM atoms does not reveal many significant differences for the calculated charges along the reaction when comparing US calculations with either configuration (1) or (2) (Tables S5–S8). The main difference is observed at the TS, where for Lys73:OQ1, the charge is more positive, and for DW:O, the charge is more negative for configuration (2), as expected by the shift in the TS location toward the TI.

For MER, the lowest calculated deacylation barrier is 11.2 kcal/mol with an average of 2.4 (±0.1) and 1.4 (±0.0) hydrogen bonds accepted by K73:OQ1 and OQ2, respectively. This barrier is 2.8 kcal/mol higher than the lowest calculated barrier for IME or 2.2 kcal/mol including the free energy penalty (derived from MM MD for IME) for orientation I. In contrast to IME, the hydroxyl of the 6α-hydroxyethyl moiety in MER always rotates during unrestrained US to hydrogen bond configuration (2), donating a hydrogen bond to the DW. This rotation occurs before the TS is reached even when configuration (1) is present in the starting structure. Enforcing the donation of a hydrogen bond from the DW to 6α-hydroxyethyl −OH, that is, restraining the reaction simulations to configuration (1), affects the location of the TS in a similar manner to that observed with IME. TS locations for configurations (1) and (2) are at −0.2/1.8 and −0.5/2.0 Å (proton transfer/nucleophilic attack), respectively. However, changing the hydrogen bonding pattern between configurations has only a minimal effect on the energetics as the barrier for (1) is 11.9 kcal/mol. Therefore, the decrease in activation energies for configuration (1) versus (2) does not follow the same trend for MER as it does for IME. A possible reason for this may be the presence of a 1β-methyl group in MER as this may hinder the rotation of the 6α-hydroxyethyl group to better optimize further hydrogen bonds between active site residues and water molecules nearby. Such a hindrance of 6α-hydroxyethyl rotation may also explain the preference observed for configuration 2 as the DW approaches the acyl carbon. A water molecule lodged between Tyr211 and Thr213 accepts a hydrogen bond from the carbapenem −OH moiety in configuration (1) or donates a hydrogen bond to it in configuration (2) (Figures 5 and S8). The 1β-methyl group occupies the space above this water and may therefore induce its displacement or the reorganization of the surrounding water molecules to optimize hydrogen bonds between them, which could subsequently lead to a change from configuration (1) to (2). Additionally, the initial nucleophilic approach of the DW (from 3.5 to 2.2 Å) with the 6α-hydroxyethyl moiety in orientation I and hydrogen bond configuration (1) is calculated to be slightly lower in energy for IME (Figure S9). The DW remains hydrogen bonded to the hydroxyethyl oxygen during this approach, with the average distance to the hydroxyethyl methyl carbon reducing to about 3.3 Å. Notably, the initial approach between the DW and the carbapenem is also slightly higher in energy in orientations II and III than in orientation I, which may contribute to their overall energetics being less favorable for deacylation. However, the reasons for the preference for the IME, but not the MER, complex to adopt configuration (1) during deacylation are likely subtle and can result from small structural changes between the active site, substrate, and solvent molecules.

### Comparison with Experimental Data

Most of the variants in the OXA-48 family are carbapenemases, with elevated IME hydrolysis rates when compared against other carbapenems.^59^ For OXA-48, experimental measurements of *k*_cat_ values for IME hydrolysis vary between 1.5 and 22.5 s^–1^, which can be converted to free energy barriers for activation (Δ^⧧^G) from 15.7 to 17.3 kcal/mol using the Eyring equation. For MER, the measured *k*_cat_ values range between 0.07 and 0.16 s^–1^, which converts to barriers from 18.7 to 19.2 kcal/mol. Using these figures as experimental estimates of free energies of activation, the difference (ΔΔ^⧧^*G*) between IME and MER hydrolysis is between 1.4 and 3.5 kcal/mol, which is approximately the same magnitude as the strength of a single hydrogen bond (1–3 kcal/mol).^60^ Hence, structural factors contributing to more efficient breakdown of IME, compared to 1β-methyl carbapenems, are most likely to be subtle. Our QM/MM simulations suggest that orientation I of the 6α-hydroxyethyl group is the most likely AC orientation to undergo deacylation, when this exists in a state with decreased hydration around Lys73:OQ2 (i.e., with only one water molecule donating a hydrogen bond to this carboxylate oxygen). When comparing the lowest free energy barriers calculated in orientation I for IME and MER (Figure 3), the difference (ΔΔ^⧧^*G*) for the two substrates is 2.8 kcal/mol; including the free energy penalty for the IME 6α-hydroxyethyl moiety adopting orientation I (0.6 kcal/mol, as determined from our MM MD simulations), the obtained ΔΔ^⧧^*G* value drops to 2.2 kcal/mol. This is in excellent agreement with the experimentally determined range of ΔΔ^⧧^*G* values. This strongly supports our assumption that TI formation is the rate-limiting process for carbapenem hydrolysis by OXA-48, consistent with similar findings for ceftazidime breakdown by OXA-48-like enzymes^35^ and carbapenem breakdown by a range of class A SBLs.^41,42^ The agreement further implies that the difference between IME and MER deacylations in OXA-48 may indeed be caused by the subtle difference in the preferred hydrogen bonding patterns involving the DW and the 6α-hydroxyethyl side chain reported here. In turn, the presence of the MER 1β-methyl group apparently contributes to this difference by influencing both the orientation of the 6α-hydroxyethyl group and the organization of water molecules in the near vicinity. (We further note that the underestimation of the absolute barriers can be fully accounted for by comparison of DFTB2 to higher level QM calculations, which indicates that DFTB2 underestimates barriers by ∼6.3–8 kcal/mol, see Table S4 and Figure S11. Thus, combined with the free energy penalty of 0.6 kcal/mol noted above, the corrected lowest barriers would be 15.3–17.0 and 17.5–19.2 kcal/mol for IME and MER, respectively.) Based on our MD simulations, the carbapenem tail groups are highly flexible and are thus unlikely to directly affect deacylation efficiency. Differences in *k*_cat_ (reflecting the rate-limiting deacylation step) for carbapenems might therefore be explained similarly to our findings here, with differences largely caused by the presence or absence of the 1β-methyl group. This is consistent with experimental data for OXA-48, which show higher *k*_cat_ values for IME and panipenem versus 1β-methyl containing carbapenems.^32,61^

Overall, our analysis of the effects of active site conformations on carbapenem hydrolysis activity highlights the importance of controlling water access to the active site. On the one hand, it is crucial for the enzyme active site to support the binding of the DW (through the aforementioned water channel). On the other hand, partial desolvation of the catalytic base (carboxylated Lys73) is required for efficient reaction. Such intricate control of active site solvation is a common feature of enzyme activity. For example, in ketosteroid isomerase, additional water molecules hydrogen bonding to the catalytic aspartate raise the barrier of the reaction significantly.^62^ Notably, this increased solvation occurs through water molecules hydrogen bonding to the carboxylate oxygen that is not receiving the proton, similar to what is observed here (difference between high and low barriers in Figure 3) but different from what we observed for ceftazidime hydrolysis.^36^ Such additional hydrogen bonding will decrease the p*K*_a_ of the catalytic carboxylate base,^63−65^ weakening its proton affinity and thereby leading to higher barriers for the reaction. To avoid or limit the occurrence of additional hydrogen bonding to catalytic bases, enzymes have evolved active site architectures that can promote desolvation to increase carboxylate reactivity. Such desolvation can, for example, be achieved by loop closure (as in triosephosphate isomerase and dihydrofolate reductase)^66,67^ or closure of the substrate binding cleft (as in ketosteroid synthase). Here, subtle control of the solvation around the carboxylated Lys73 is related to nearby hydrophobic residues (Val120 and Leu158), which can adopt conformations that allow the presence of the DW but avoid more extensive solvation of the catalytic carboxylate.

## Conclusions

We have modeled carbapenem hydrolysis by the OXA-48 β-lactamase using QM/MM reaction simulations. The deacylation reaction was modeled for two carbapenem substrates, IME and MER, to deduce the origin of the higher activity toward IME compared to other carbapenems. MM MD simulations of the AC complexes demonstrate that the carbapenem tail (C2) groups are able to adopt many different conformations. In contrast, the carbapenem 6α-hydroxyethyl group is able to rotate and to adopt three specific different orientations, where it either interacts with the DW (I) or with Lys73 (III) or is rotated so that the methyl group is oriented toward Leu158 (II). Subsequently, deacylation was modeled using QM/MM for both substrates in these three orientations to investigate the effect of orientation upon deacylation efficiency. Our calculated free energy barriers indicate that the most deacylation-competent orientation is I, where the hydroxyl group interacts with the DW, and that orientation III leads to the highest free energy barriers.

Detailed comparison of the simulations revealed two factors that significantly affect the reaction energetics: hydration around Lys73 and the hydrogen bonding pattern between the DW and substrate, specifically the 6α-hydroxyethyl group. Hydration around the general base has been proposed to affect the predicted hydrolysis rates for other β-lactam substrates;^36^ here, we show that this is affected by hydration around both Lys73 carboxylate oxygens (not only the oxygen participating in proton transfer). Increased hydration around the nonreactive oxygen (Lys73:OQ2) correlates with higher calculated barriers; in turn, the orientation of Val120 correlates with the number of water molecules near this oxygen. Another aspect influencing the deacylation efficiency is the pattern of hydrogen bonds in the active site that involve the DW and the carbapenem 6α-hydroxyethyl side chain. IME shows a preference for a configuration in which the DW donates hydrogen bonds to Lys73 and the 6α-hydroxyethyl hydroxyl group; the free energy barrier is higher when the hydroxyl group instead rotates to donate a hydrogen bond to the DW. This preference is not observed for MER: simulations with both hydrogen bond configurations have comparable energy barriers, which are similar to that calculated for IME in the less favorable orientation. Therefore, we can conclude that the difference between hydrolytic activities for the two carbapenem substrates stems from subtle differences in the active site hydrogen bonding patterns, which affect the reactivity of the DW. Furthermore, our results indicate that active site hydration is an important determinant of catalysis in OXA-48 enzymes: increasing hydration around the general base impairs carbapenem hydrolysis. Our study highlights the importance of detailed atomistic modeling in addition to experimental research to determine the exact origins of catalytic activity. Simulation protocols such as those employed here can extend information from crystallographic studies to enable investigation of the strength and dynamics of specific active site interactions during the catalytic cycle and directly investigate determinants of activity *in situ*.

## Supporting Information Available

Simulation setup and analysis; cluster analysis of AC structures; Leu158 and V120 rotamer sampling; full results of FESs in different conformations; analysis of the DW approach; benchmarking details; and Mulliken charge analysis (PDF)
Simulation parameter files; input files; starting structures; and TS structures/ensembles (ZIP)

## Abbreviations

SBL: serine β-lactamase
MBL: metallo-β-lactamase
AC: acylenzyme
TS: transition state
TI: tetrahedral intermediate
QM/MM: quantum mechanics/molecular mechanics
US: umbrella sampling
FES: free energy surface
