# Supplementary material for: Multiscale Simulations Identify Origins of Differential Carbapenem Hydrolysis by the OXA-48 β-Lactamase
Source: ACS Catal. 2022 Apr 4;12(8):4534–44. doi: 10.1021/acscatal.1c05694 (PMC9097296; doi:10.1021/acscatal.1c05694)
Supplement: Supplementary file 1 — cs1c05694_si_001.pdf [file cs1c05694_si_001.pdf]

## Supporting Information

# Multiscale Simulations Identify Origins of Differential Carbapenem Hydrolysis by the OXA-48 β-lactamase

*Viivi H. A. Hirvonen[a,b], Tal Moshe Weizmann[a,†], Adrian J. Mulholland[b], James Spencer[c] and Marc W. van der Kamp[a,b]**

[a] School of Biochemistry, University of Bristol, University Walk, Bristol, BS8 1TD, UK; marc.vanderkamp@bristol.ac.uk

[b] Centre for Computational Chemistry, School of Chemistry, University of Bristol, Cantock's Close, Bristol, BS8 1TS, UK.

[c] School of Cellular and Molecular Medicine, University of Bristol, University Walk, Bristol, BS8 1TD, UK.

## System Setup

The OXA-48 with imipenem model was set-up starting from a corresponding acylenzyme (AC) crystal structure (PDB ID: 6P97)[1], as well as OXA-48 with meropenem (PDB ID: 6P98)[1]. In both structures, Lys73 is not carboxylated and was thus replaced by its carboxylated form as found in another OXA-48 + imipenem acylenzyme structure (PDB ID: 5QB4)[2]. The deacylating water (DW) was added manually to both models, and all crystallographic water molecules were kept in place. The p$K_a$ values of titratable residues were inspected using propKa 3.1,[3] and all residues were kept in their standard state (protonated Lys, deprotonated Glu and Asp residues). Histidine tautomers were assigned using the reduce program (from AmberTools): all histidines were singly protonated at the ε-nitrogen except for residues 38 and 140, which were singly protonated at the δ-nitrogen. Hydrogens were added to the structures using tLeap upon system preparation, and the enzyme was solvated in a TIP3P water box extending at least 12 Å from the enzyme. The systems were neutralized by randomly replacing bulk water molecules with $Na^+$ counter ions (using the default TIP3P compatible ion parameters in Amber). The ff14SB parameter set was used for the protein.[4]

Partial charges and force field parameters for the non-standard Ser70+carbapenem residues were derived using the restrained electrostatic potential (RESP) fitting as implemented in the R.E.D. Server.[5] Analogous gaff parameters were substituted for any missing parameters.[6] Parameter files for carboxylated lysine (as used previously in ref. [7]) and carbapenem ACs are available as part of the Supporting Information.

## Computational Methods

After system preparation, all models were initially briefly minimized for 2000 steps to avoid any steric clashes (1000 steps conjugate gradient and 1000 steps steepest descent). After minimization, the systems were heated from 50 K to 300 K in 20 ps, and subsequently simulated for 200 ns in the NPT ensemble. Langevin dynamics were used in all simulations with a collision frequency of 0.2 $ps^{-1}$. MD timestep was 2 fs, and all bonds involving hydrogen atoms were constrained using the SHAKE algorithm. Structures during MD were recorded every 20 ps (10k MD frames/trajectory). The Amber18 package with Ambertools19 was used for all calculations,[8] and specifically the pmemd.cuda MD engine for all extended MM MD simulations.[9-11] All trajectory analysis was done using cpptraj[12] (as implemented in Ambertools19) excluding 50 ns from the start to allow time for system equilibration. Hydrogen bond analysis using cpptraj was done using the default criteria set in cpptraj (donor-acceptor distance less than 3.4 Å, the D-H-A angle between hydrogen bond donor and acceptor deviates less than 45° from a linear angle).

Starting structures for QM/MM umbrella sampling (US) were chosen from restart files saved during the 200 ns MM MD simulations based on visual inspection (starting structures taken at least 1 ns apart unless otherwise stated). All starting snapshots had the desired 6α-hydroxyethyl orientation and a suitable DW in place, as to not introduce big distortions through restraints when starting US calculations. Three different starting structures were used per free energy barrier (unless otherwise stated). The DFTB2 (SCC-DFTB)[13, 14] method was used for describing the QM region consisting of either 43 or 46 atoms for imipenem and meropenem, respectively (Figure S1). The ester bond in the AC was restrained with a one-sided harmonic restraint to avoid elongation beyond 1.6 Å (force constant 100 kcal $mol^{-1}$ $Å^{-2}$), and the 6α-hydroxyethyl group dihedral was restrained near its initial values during reaction simulations. In orientation II, the sidechain dihedral was restrained between values 150-200°, and in

orientation III between 270-310°; the applied force constant was 50 kcal mol$^{-1}$ Å$^{-2}$. No dihedral restraints were applied in orientation I, as switching from this orientation to another one was not observed during US. However, distance restraints between the meropenem 6α-hydroxyethyl hydroxyl group and an active site water molecule were applied to avoid drifting away from the active site configuration where the DW donates a hydrogen bond the 6α-hydroxyethyl group. A one-sided harmonic restraint was added for this hydrogen bond to avoid elongation beyond 2.2 Å (force constant of 50 kcal mol$^{-1}$ Å$^{-2}$).

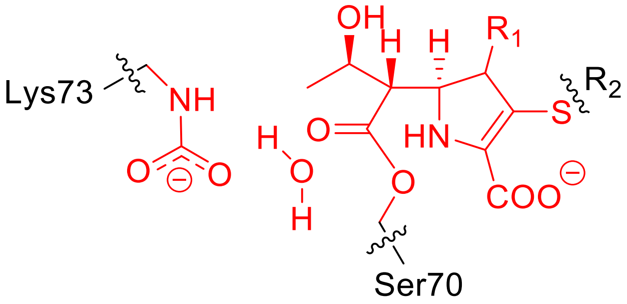


*Figure S1. QM region in US simulations, QM atoms in red. Link atoms based along bonds indicated with wavy lines.*

Full deacylation free energy surfaces (FESs) were calculated using two reaction coordinates to describe the proton transfer (PT) and nucleophilic attack (NA). The PT reaction coordinate is defined as a combination of the forming K73:O – DW:H and the breaking DW:O – DW:H distance (*d*(K73:O-DW:H)-*d*(DW:O-DW:H)), and the NA reaction coordinate as the distance for the forming DW:O – C bond (*d*(DW:O – C)). Reaction coordinate values were decreased from 0.8 Å /3.5 Å (PT/NA) in the AC to −1.0 Å /1.5 Å in the TI by 0.1 Å. Force constants for both reaction coordinates were 100 kcal mol$^{-1}$ Å$^{-2}$. Full deacylation free energy surfaces (FESs) were constructed by first performing US along an approximate diagonal on the FES (used as a proxy for a likely minimum free energy path), and then calculating the rest of the US windows using these calculations as a starting point. Initial sampling along the diagonal included 36 windows with values:

| **PT** | 0.8 | 0.8 | 0.8 | 0.8 | 0.8 | 0.8 | 0.8 | 0.8 | 0.7 | 0.7 | 0.7 | 0.6 | 0.6 | 0.5 | 0.5 | 0.5 | 0.5 |
|---|---|---|---|---|---|---|---|---|---|---|---|---|---|---|---|---|---|
| **NA** | 3.5 | 3.4 | 3.3 | 3.2 | 3.1 | 3.0 | 2.9 | 2.8 | 2.7 | 2.6 | 2.5 | 2.5 | 2.4 | 2.4 | 2.3 | 2.2 | 2.2 |

| | | | | | | | | | | | | | | | |
|---|---|---|---|---|---|---|---|---|---|---|---|---|---|---|---|
| **PT** | 0.4 | 0.3 | 0.2 | 0.1 | 0.0 | -0.1 | -0.2 | -0.3 | -0.3 | -0.4 | -0.5 | -0.6 | -0.7 | -0.7 | -0.7 |
| **NA** | 2.2 | 2.2 | 2.1 | 2.1 | 2.1 | 2.1 | 2.1 | 2.1 | 2.0 | 2.0 | 1.9 | 1.9 | 1.9 | 1.9 | 1.8 |

| | | | | | | |
|---|---|---|---|---|---|---|
| **PT** | -0.7 | -0.8 | -0.8 | -0.8 | -0.9 | -1.0 |
| **NA** | 1.7 | 1.7 | 1.6 | 1.5 | 1.5 | 1.5 |

The whole FESs consisted of a total of 399 US windows, and the sampling time was 2 ps/window with a 1 fs timestep. FESs were constructed using the weighted histogram analysis method (WHAM, Grossfield lab)[15] with 19 and 21 bins for the PT and NA coordinates, respectively. The convergence criterion was set to $10^{-13}$. Minimum free energy paths on the FESs were analyzed using the Minimum Energy Path Surface Analysis (MEPSA) program.[16] All US calculations were performed using sander.MPI as implemented in Ambertools19.[8]

## Acylenzyme Clustering

Clustering of the AC orientations was done separately for imipenem and meropenem MM MD trajectories (excluding 50 ns from the start, five simulations per substrate with 7500 frames per simulation). Trajectories were aligned on Cα-atoms of residues 70-73, 118-120, 157, 158, and 209-211, and substrate orientations were clustered into four groups based on AC heavy atom RMSD. The kmeans algorithm (as implemented in cpptraj) was used for the clustering procedure with a sieve of 10. The representative structures for the four clusters are presented in Figure S2 along the corresponding AC orientation in the original crystal structure.

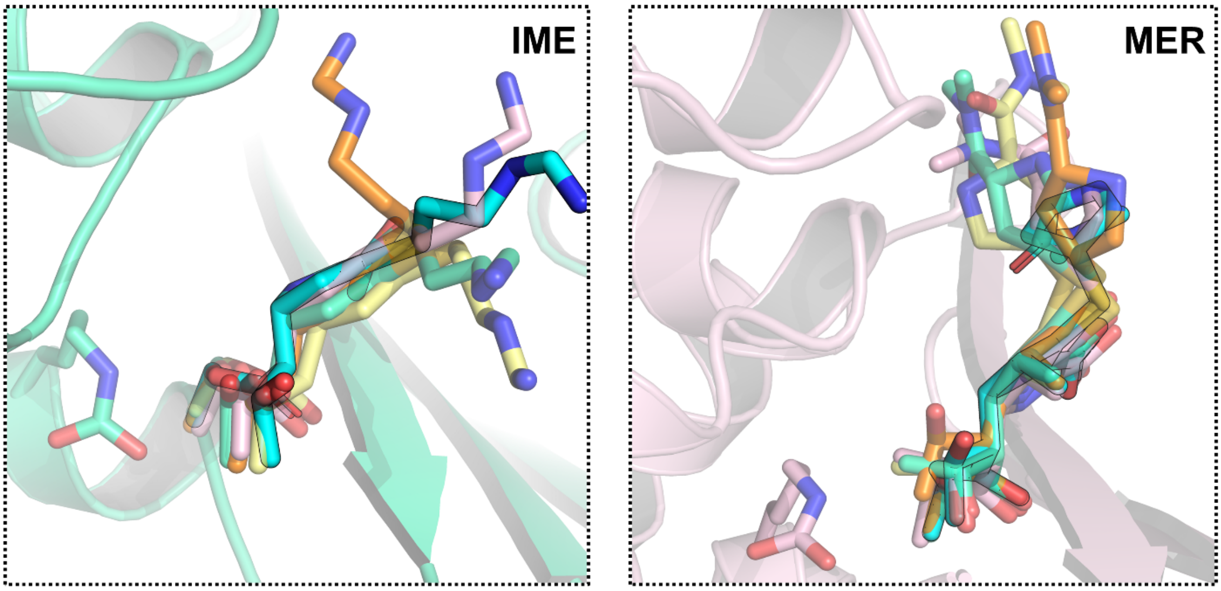


*Figure S2. Representative structures from AC clustering for imipenem (left) and meropenem (right). Cluster 1, green; cluster 2, pink; cluster 3, yellow; cluster 4, orange; the crystallographic binding pose in cyan.*

*Table S1. Clustering of the substrate binding pose for imipenem (IME) and meropenem (MER). Fraction represents the fraction of simulation frames belonging to the clusters and colour the corresponding carbon atom colouring in Figure S2. RMSD measured between carbapenem heavy atoms in the representative cluster structure and in the crystal structure.*

| | | **IME** | | **MER** | |
|---|---|---|---|---|---|
| | Colour | Fraction | RMSD (Å) | Fraction | RMSD (Å) |
| Cluster 1 | Green | 0.55 | 0.81 | 0.36 | 1.65 |
| Cluster 2 | Pink | 0.21 | 0.82 | 0.34 | 1.75 |
| Cluster 3 | Yellow | 0.13 | 1.09 | 0.25 | 2.16 |
| Cluster 4 | Orange | 0.11 | 1.75 | 0.05 | 2.49 |

In addition to AC clustering, the active site conformations for both imipenem and meropenem simulations were compared by combining all trajectories and clustering the structures based on the common AC atoms (i.e. all atoms except C2 tail groups beyond sulphur and the 1β-group). Solvent molecules, counterions, and parts of the enzyme which were not identical between the two models were stripped before clustering. Trajectories were aligned on the mainchain heavy atoms of residues 70, 73, and 157, and clustering done based on RMSD

of the remaining carbapenem heavy atoms. The kmeans clustering algorithm was used to divide the structures in to four clusters with a sieve of 10.

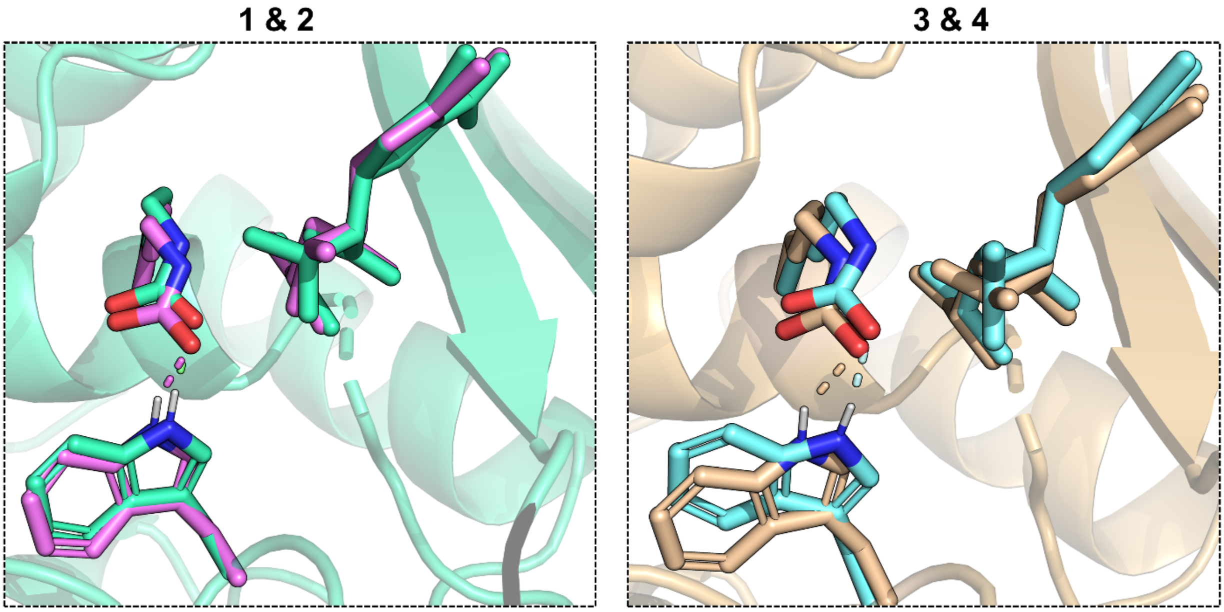


*Figure S3. Representative cluster structures for the combined clustering of imipenem and meropenem trajectories. Left: Clusters 1 and 2 are sampled for most of the simulation time for meropenem (green) and imipenem (purple), respectively. Right: For the rest of time, cluster 3 (brown) is sampled for imipenem and cluster 4 (cyan) for meropenem.*

*Table S2. Clustering of the common carbapenem scaffold in imipenem and meropenem trajectories. Fraction represents the fraction of simulation frames belonging to the clusters and colour the corresponding carbon atom colouring in Figure S3.*

| | Color | Fraction (IME) | Fraction (MER) |
|---|---|---|---|
| Cluster 1 | Green | 0.00 | 0.71 |
| Cluster 2 | Purple | 0.65 | 0.00 |
| Cluster 3 | Brown | 0.35 | 0.00 |
| Cluster 4 | Cyan | 0.00 | 0.29 |

## Leu158 Dihedral Sampling

Leu158 has been proposed to modulate the access of bulk solvent near Lys73 in ceftazidime deacylation.[7] The orientations of Leu158 were analysed by measuring the sidechain $\chi_1$ dihedral angle (N-Cα-Cβ-Cγ). In both crystal structures used for model building, Leu158 is in the *g*– orientation in the starting structures. (The $\chi 2$ dihedral (Cβ - Cγ bond) is 170° and 91° for the imipenem and meropenem AC structures, respectively.[1]) Upon MD equilibration, Leu158 almost always adapts to the *t* orientation (Figure S4).

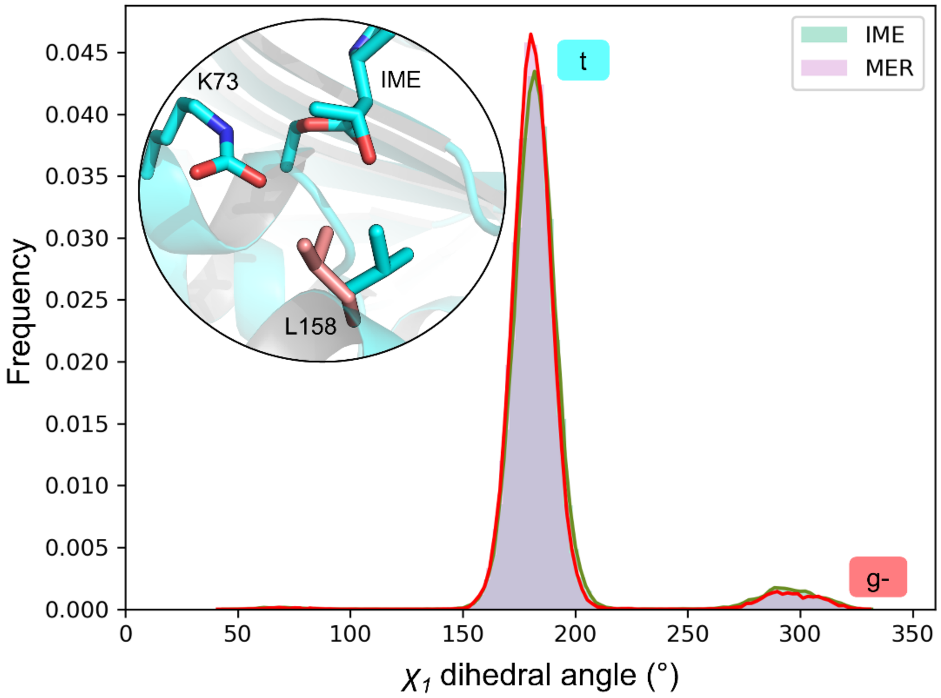


*Figure S4. Sampling distribution for Leu158 rotamers in MM MD for imipenem (green) and meropenem (red). Leu158 changes from the starting g– orientation (red sticks representation in the insert) to the t orientation (cyan) for the majority of the simulation time.*

## Nucleophilic attack distance

As a preliminary indication of the effect of a certain AC conformation on the likelihood of nucleophilic attack, the distance between the AC electrophilic carbon and the closest water molecule was measured. The corresponding scatter plots are presented in Figure S5, where a

threshold of 4 Å is indicated with a dashed line; distances below this threshold are regarded suitable for a nucleophilic attack (although the choice of this threshold value is somewhat arbitrary). For both carbapenems, a water molecule is found at a suitable distance for the nucleophilic attack in all three orientations, even when many active site configurations fall beyond 4 Å for orientations II (~180°) and III (~290°).

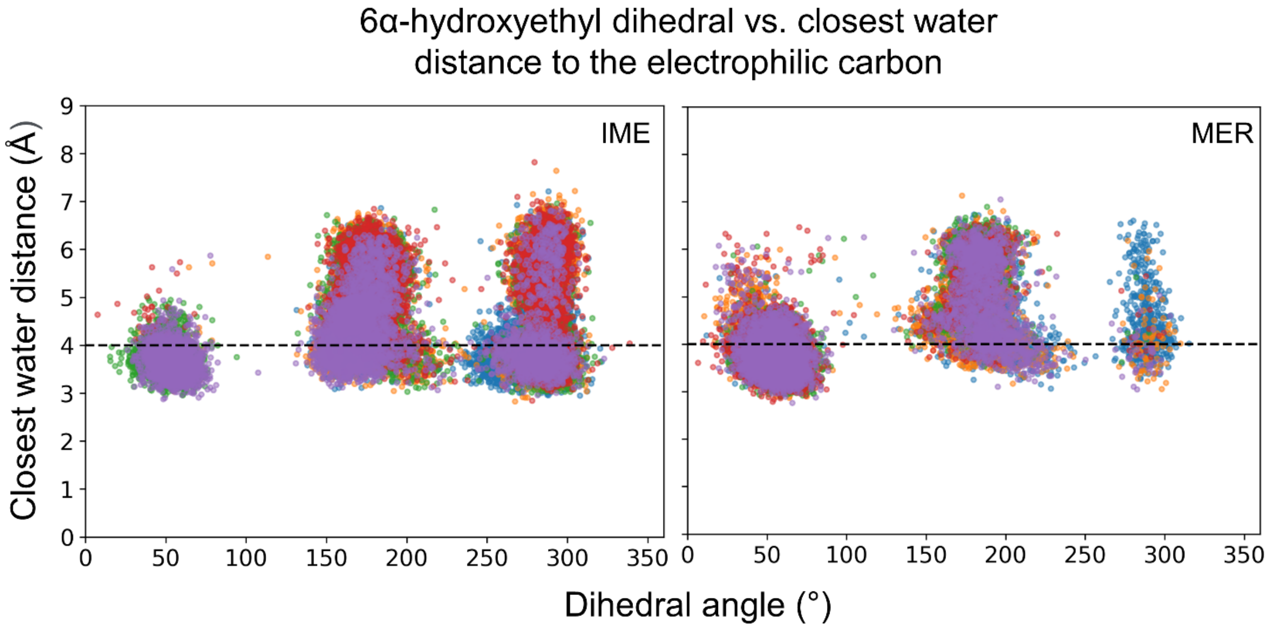


*Figure S5. The distribution of distances from the AC electrophilic carbon to the closest water molecule in the active site. Distances measured for all five simulations per system (50 ns were excluded from the start for each simulation, total simulation length 200 ns). Each scatter point represents a frame in a trajectory, different colours represent different trajectories. Dashed line at 4 Å is used as an arbitrary threshold distance for indicating the feasibility of nucleophilic attack, i.e. arrangements beyond 4 Å are not likely to undergo efficient deacylation.*

## Val120 Dihedral Sampling

Val120 adopts three different orientations during MM MD, which can be differentiated by the $\chi_1$ (N-Cα-Cβ-Cγ1) dihedral angle: *g*+ at ~60°, *t* at ~180°, and *g*– at ~300°. Val120 is situated in the vicinity of Lys73 and Trp157, and its rotamer influences the accessibility of water near Lys73 (especially near Lys73:OQ2). The distribution of Val120 $\chi_1$ dihedrals

sampled is presented in Figure S6. The *t* rotamer, which is observed in both crystal structures used as starting models, is the most sampled state for both imipenem and meropenem; this orientation allows for two water molecules to hydrogen bond with Lys73:OQ2. When the dihedral angle rotates to the *g*+ state, only one water has space to donate a hydrogen bond to OQ2, as the γ2-carbon occupies the space available for water in the case of *t* rotamer. The *g*– rotamer was observed the least, and it was not present in any starting structure used for US.

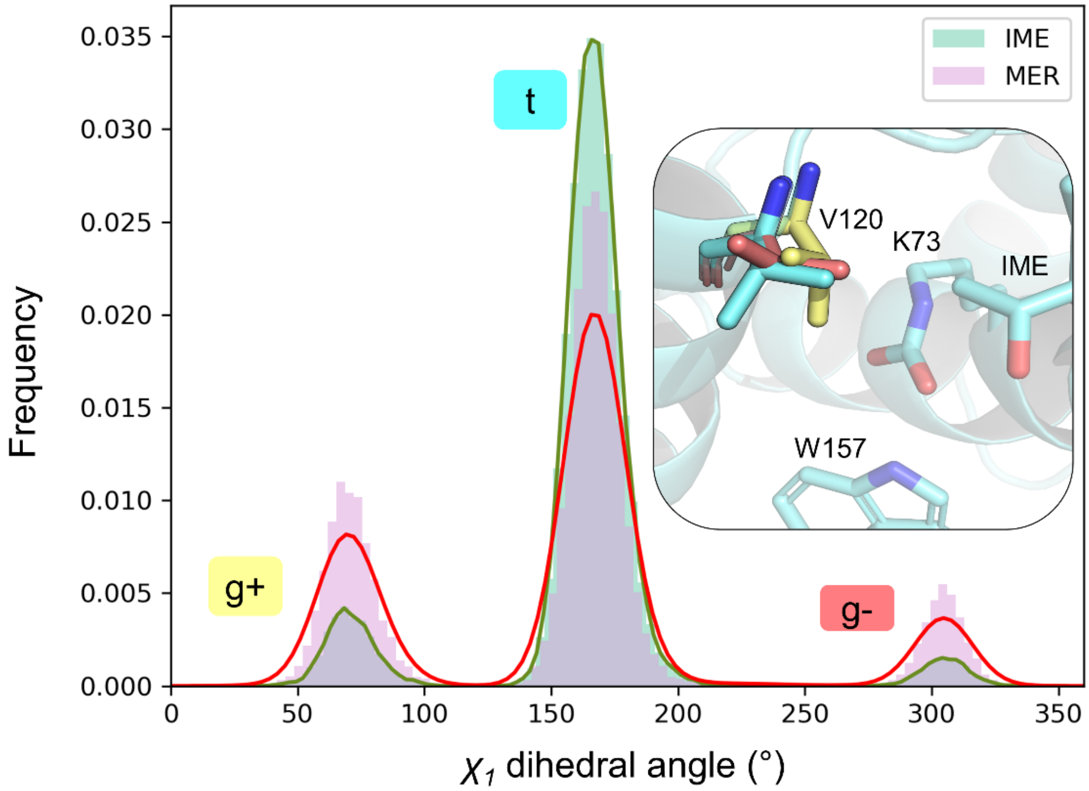


*Figure S6. V120 rotamer distribution in MM MD for imipenem (green) and meropenem (red). Rotamer label colouring corresponds to the stick colour representation of the rotamer structure in the inset. Histograms created using 100 bins, probability distributions (solid line) obtained using the kernel density estimation in the seaborn library (python) with default parameters.*

## Deacylation Umbrella Sampling Results

Free energy barriers of the simulated (rate-limiting) first step of deacylation for different AC orientations with different hydration states are presented in Table S3. Barriers are calculated by combining sampling from three separate US calculations into one WHAM

calculation (the overall sampling time being 3x2ps = 6ps/window, corresponding to 2.4 ns for the full surface), standard deviations are between the individual snapshots.

*Table S3. Free energy barriers for carbapenem deacylation from umbrella sampling. All energies in kcal/mol, standard deviations between barriers obtained for the three individual US calculations in parenthesis. Hydration state 1 = only one water donating a hydrogen bond to Lys73:OQ2, two waters donating a hydrogen bond to Lys73:OQ1, hydration state 2 = two waters hydrogen bonded to both Lys73 carboxylate oxygens.*

| | Imipenem | | Meropenem | |
|---|---|---|---|---|
| | Hydration state 1 | Hydration state 2 | Hydration state 1 | Hydration state 2 |
| Orientation I[1] | 8.4 (0.9) | 10.4 (0.4) | 11.2 (0.9) | - |
| Orientation I[2] | 10.4 (0.4) | 13.5 (1.0) | 11.9 (0.6) | 15.3 (1.1) |
| Orientation II | 10.5 (1.1) | 13.6 (0.9) | 13.6 (0.9) | 16.0 (1.1) |
| Orientation III | 11.2 (0.6)[3] | 20.8 (1.4) | 12.4 (2.0)[3] | 18.0 (0.5) |

[1] DW donates a hydrogen bond to the carbapenem hydroxyl group

[2] DW accepts a hydrogen bond from the carbapenem hydroxyl group

[3] Only one water is donating a hydrogen bond to Lys73:OQ1; when a second water hydrogen bonds to OQ1, barriers increase to ~15 kcal/mol for both imipenem and meropenem (fewer than three US runs each as this conformation is rare; results not shown).

The FESs for both imipenem and meropenem for all three 6α-hydroxyethyl orientations are presented in Figure S7. The minimum free energy paths are illustrated with black dots. All surfaces are for the active site configuration where only one water donates a hydrogen bond to Lys73:OQ2. In orientation I for imipenem, the DW donates a hydrogen bond to the 6α-hydroxyethyl hydroxyl group, whereas for meropenem, the DW accepts a hydrogen bond from this hydroxyl. For some meropenem simulations, additional restraints were placed on bonds involving hydrogens in the QM region to prevent unwanted proton transfers during reaction simulations.

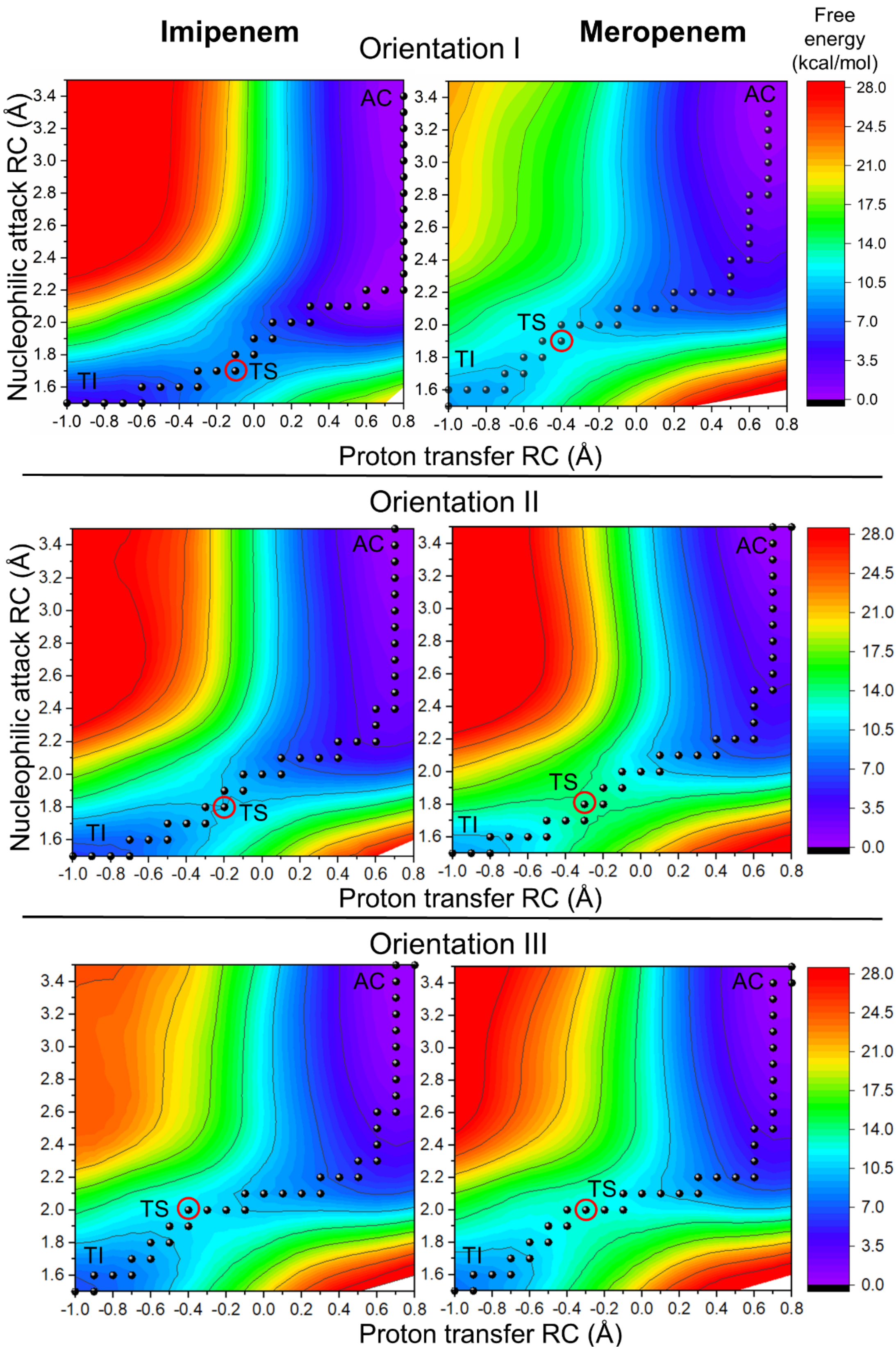


*Figure S7. Calculated free energy surfaces for imipenem (left) and meropenem (right) deacylation in substrate orientations I (top), II (middle), and III (bottom)s. The minimum free energy paths marked with black dots. AC=acylenzyme, TS=transition state (circled), TI=tetrahedral intermediate.*

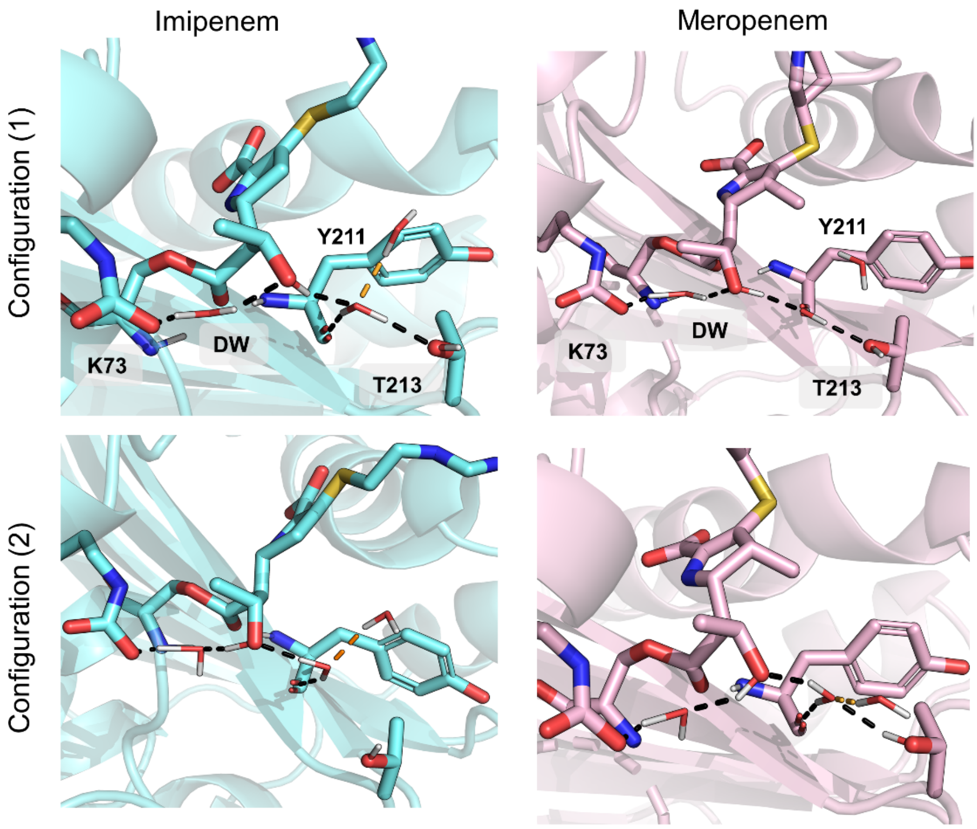


*Figure S8. Transition state structures for imipenem and meropenem in orientation I. In configuration I (top row), the DW donates a hydrogen bond to the 6α-hydroxyethyl group, which in turn donates a hydrogen bond to a water molecule between Tyr211 and Thr213. For imipenem, this water can accept an additional hydrogen bond from another water molecule near the imipenem 1β-proton. For meropenem, this additional water is shifted due to presence of the 1β-methyl group.*

To further inspect the possible influence of the initial approach of the DW to the carbapenem electrophilic carbon on the observed difference in efficiency between imipenem and meropenem, this approach was inspected by QM/MM US simulations where only the distance between the DW oxygen and the carbapenem carbon was used as a reaction coordinate. This was done for both imipenem and meropenem in active site configuration (1), where the DW donates a hydrogen bond to the 6α-hydroxyethyl group. Based on the calculated free energies, the energetic cost for the initial approach of the DW is significantly lower for imipenem than for meropenem, which partly explains the lower calculated barriers in imipenem deacylation (Figure S9, left y-axis). Additionally, the distances between the DW:O and the methyl carbon or hydroxyl oxygen in the 6α-hydroxyethyl group were recorded for the

extended 20 ps US runs. The distances were averaged to yield one value per US window (Figure S9, right y-axis).

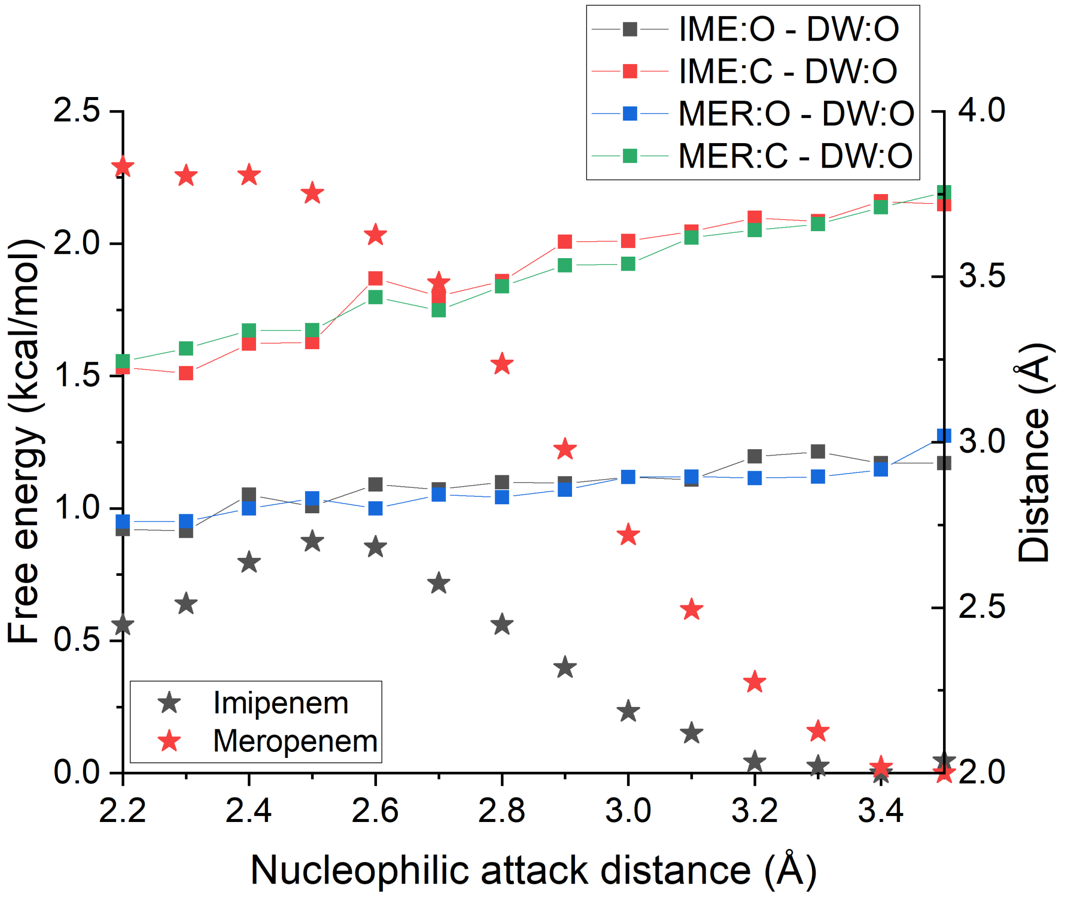


*Figure S9. Left y-axis: The calculated free energy profile for the initial approach of the deacylating water molecule to the carbapenem electrophilic carbon (depicted as a scatter plot with stars). Sampling was performed for 20 ps/window for each of three snapshots starting from structures obtained from the full 2 ps free energy surface. Right y-axis: Average distances between the deacylating water oxygen and either the 6α-hydroxyethyl hydroxyl oxygen (IME/MER:O) or the 6α-hydroxyethyl methyl carbon (IME/MER:C) in the extended 20 ps US calculations.*

## Benchmarking

DFTB2 (SCC-DFTB) was used as the QM method in all US simulations. Previously, this method has been shown to depict deacylation in serine β-lactamases well and to distinguish between carbapenemases and carbapenem-inhibited class A enzymes.[17] For OXA-48, the minimum free energy paths on DFTB2//ff14SB free energy surfaces indicate that the proton transfer and nucleophilic attack occur in a concerted fashion after the initial approach of the

DW (from ~3.5 Å to 2.2 Å). The lowest calculated free energy barrier, which we expect to be a representative barrier for the most efficient deacylation reaction, was 8.5 kcal/mol, which is a significant underestimation from the experimental barrier of 16.6 kcal/mol (converted from a $k_{cat}$ of 5 $s^{-1}$ using the Eyring equation)[18]. To benchmark the performance of DFTB2, we performed transition state optimization for a representative small molecule model in the gas-phase and calculated a representative deacylation potential energy surface using a generally accurate hybrid DFT functional, M06-2X/6-31+G(d), with single point energy corrections (SCS-MP2/aug-cc-pVTZ).

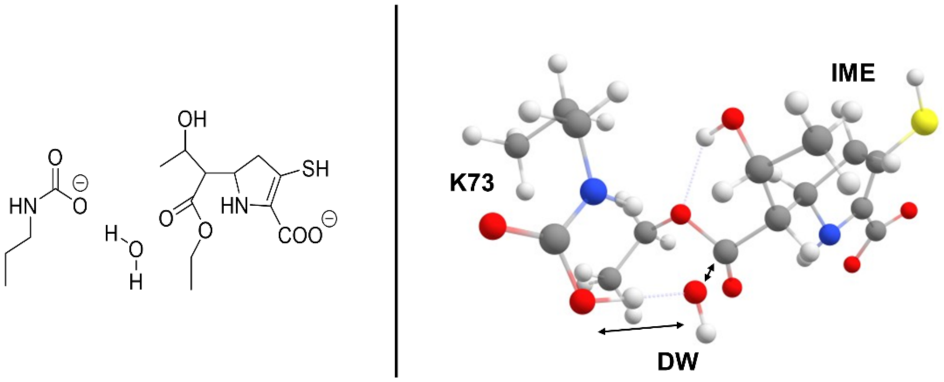


*Figure S10. Small molecule model of the active site used in benchmarking calculations. The transition state (right) corresponds to a concerted reaction, with proton transfer between the K73 carboxylate and water, and C-O bond formation between water and the carbapenem substrate (both indicated with arrows). In the gas-phase, the 6α-hydroxyethyl group has been optimized to orientation III (with a ~290° dihedral).*

The small molecule model used in benchmarking is presented in Figure S10. The gas-phase model included methyl-capped Lys73 and imipenem acylenzyme with the DW taken from a representative full enzyme structure. TS corresponding to the tetrahedral intermediate (TI) formation in deacylation was first optimized using Gaussian16[19] on the M06-2X/6-31+G(d) level using looser criteria (with keywords Opt=(TS,calcfc,noeigentest)), and the final structure was optimized with tighter convergence criteria (Opt=(TS,VTight) SCF=Tight). The ultrafine integration grid was used in all calculations (Int=UltraFine). TS was validated by visual inspection and normal mode calculation (one imaginary frequency at –296.63 corresponding to the reaction coordinates). The optimization and frequency output is included in the Supporting Information ZIP file. The reactant (AC) and product (TI) structures were obtained with IRC calculations in both directions starting from the optimized TS, and the final

IRC endpoints were further optimized on the same level as the final TS. TS optimisation and subsequent IRC calculations were done using Gaussian16.[19] Single-point energies were calculated on the higher SCS-MP2/aug-cc-pVTZ level[20, 21] (using the RIJK approximation with aug-cc-pVTZ/C and aug-cc-pVQZ/JK auxiliary basis sets), as well as using DFTB2. RI-SCS-MP2 calculations were performed with Orca 4.2,[22, 23] and semi-empirical calculations using sqm (part of the Ambertools19 package). Since the gas-phase model was optimized to the 290° orientation, the US barrier is shown for the lowest barrier with orientation III (~290°) for imipenem.

*Table S4. Benchmarking energies for the small molecule gas-phase system. The approximate TS location from US is shown for the 290° orientation as the gas-phase system was optimized to this orientation.*

| **Method** | **NA RC (Å)** | **PT RC (Å)** | **$\Delta^{\ddagger}G_{calc}$ (kcal/mol)** |
|---|---|---|---|
| M06-2X/6-31+G(d) | 1.89 | –0.37 | 19.9 kcal/mol |
| RI-SCS-MP2/aug-cc-pVTZ[a] | - | - | 22.5 kcal/mol |
| DFTB2[a,b] | - | - | 14.5 kcal/mol |
| DFTB2/ff14SB FES (OXA-48)[c] | 2.0 | -0.4 | 11.2 kcal/mol |

[a] Energy difference from single-point energies on M06-2X/6-31+G(d) optimized AC and TS structures.

[b] TS could not be optimized in the gas-phase with DFTB2 as no saddle point was indicated between the AC minimum and the tetrahedral intermediate.

[c] The approximate TS location on the QM/MM free energy surface.

To study the performance of DFTB2 in the whole enzyme model, deacylation QM/MM potential energy surfaces were calculated for imipenem in the 50° dihedral orientation using DFTB2 and M06-2X/def2-TZVP[24, 25] levels of theory for the QM region (Figure S11). The LBFGS algorithm was used for energy minimization with a convergence criterium of 0.01 $mol^{-1}$ $Å^{-1}$. Force constants for restraints (as used in US) were increased to 5000 kcal $mol^{-1}$ $Å^{-2}$, and residues further than 5 Å from the substrate were restrained (positional restraints with 50 kcal $mol^{-1}$ $Å^{-2}$ force constant). Energy correction using DFT was obtained by calculating single-point energies for the QM region on the M06-2X/def2-TZVP level (using Orca 4.2) and taking the difference between the M06-2X and DFTB2 energies (interactions terms between the QM and MM regions were thus calculated using DFTB2). The RIJCOSX approximation with the def2/J auxiliary basis set was used with M06-2X.

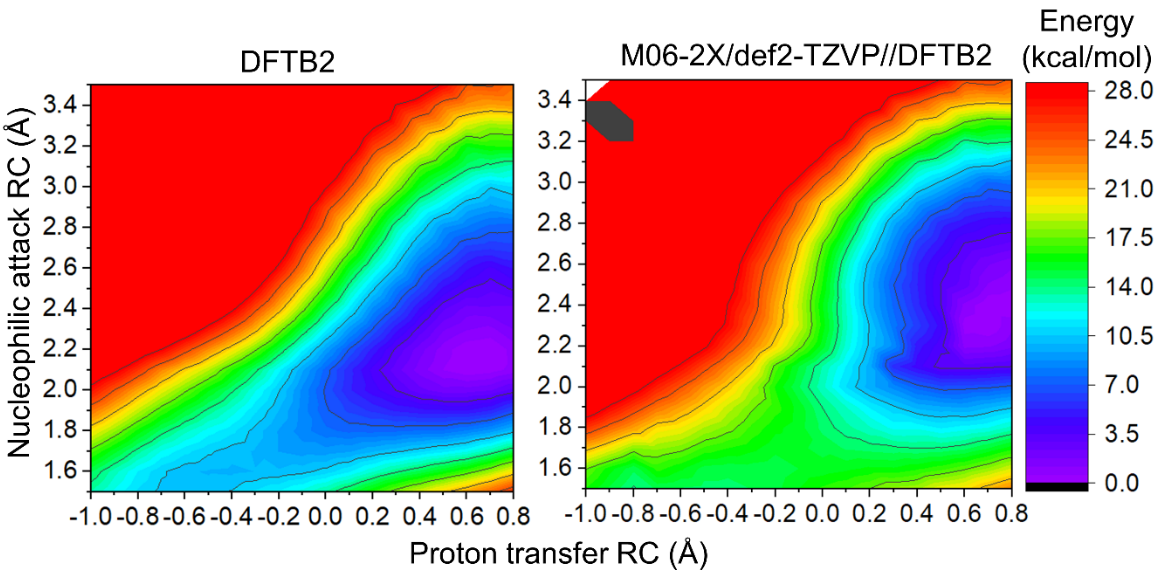


*Figure S11. QM/MM Potential energy surfaces for OXA-48/imipenem in orientation I calculated using DFTB2/ff14SB (left) or M06-2X/def2-TZVP/ff14SB (right). Energy at the approximate TS position (-0.1/1.7 for PT and NA RCs, respectively) is 9.0 kcal/mol for DFTB2, and 15.3 kcal/mol for M06-2X. SCF did not converge satisfactorily for two structures in the high energy region and were hence left out from the M06-2X potential energy surface (black area).*

## Mulliken Charges

Mulliken charges for the key atoms in the QM region were calculated from extended US at the AC, TS, and TI minima, values determined from the minimum free energy path. Each state was sampled for 20 ps (starting from the last structure after the initial 2 ps sampling), and charges were calculated every 20 fs and averaged to obtain one value per atom. Key atoms with atom labels used are presented in Figure S12.

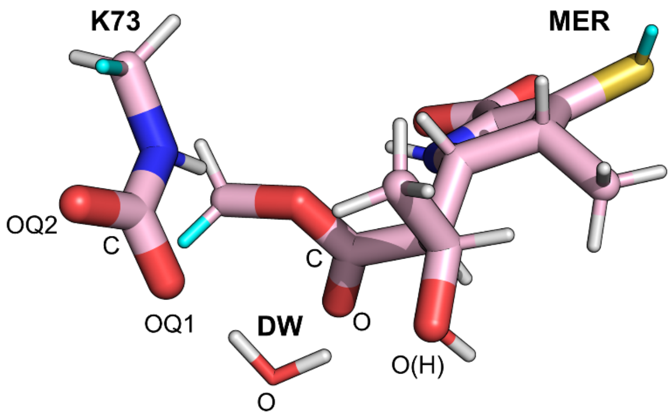


*Figure S12. QM region atoms (for meropenem) with link atoms highlighted in cyan. Names shown for the key atoms, for which Mulliken charges were recorded along the reaction.*

All Mulliken charges show the same trend: during the reaction, positive charge migrates to the Lys73 carboxylate (proton acceptor), and the substrate carbonyl oxygen becomes more negative due to negatively-charged oxyanion formation in the TI. The transition state location shifts slightly when the hydrogen bond pattern between the DW and the 6α-hydroxyethyl group changes, which results in slightly different charges at the transition state. When the DW accepts a hydrogen bond from the carbapenem hydroxyl, the transition state location shifts to more negative values for the proton transfer reaction coordinate, i.e. closer to the TI structure. This results in more positive Lys73 carboxylate oxygen charges and more negative DW:O charges. No significant changes are seen between different active site conformations when comparing the change between endpoint charges (AC → TI).

*Table S5. Mulliken charges for the key QM region atoms for imipenem in the 50° orientation. DW donates a hydrogen bond to the 6α-hydroxyethyl hydroxyl group. Locations for the minima and TS (proton transfer/nucleophilic attack values): AC 0.8/3.4, TS –0.1/1.7, TI –1.0/1.5. Standard deviation in parenthesis.*

| QM atom | **Enzyme** | | | | |
|---|---|---|---|---|---|
| | $Q^{AC}$ | $Q^{TS}$ | $Q^{TI}$ | $\Delta Q^{TS\text{-}AC}$ | $\Delta Q^{TI\text{-}AC}$ |
| K73-OQ1 | –0.84 (0.03) | –0.65 (0.03) | –0.51 (0.02) | 0.19 | 0.33 |
| K73-OQ2 | –0.76 (0.03) | –0.73 (0.03) | –0.67 (0.03) | 0.04 | 0.09 |
| K73-C | 0.74 (0.01) | 0.72 (0.01) | 0.71 (0.01) | –0.02 | –0.03 |
| DW-O | –0.69 (0.01) | –0.61 (0.03) | –0.59 (0.02) | 0.08 | 0.10 |
| IME-O | –0.60 (0.03) | –0.80 (0.04) | –0.87 (0.03) | –0.20 | –0.26 |
| IME-C | 0.66 (0.01) | 0.73 (0.02) | 0.74 (0.02) | 0.06 | 0.08 |
| IME-O(H) | –0.51 (0.02) | –0.54 (0.02) | –0.54 (0.02) | –0.03 | –0.03 |

*Table S6. Mulliken charges for the key QM region atoms for imipenem in the 50° orientation. DW accepts a hydrogen bond from the 6α–hydroxyethyl hydroxyl group. Locations for the minima and TS: AC 0.7/3.4, TS –0.4/1.9, TI –1.0/1.5. Standard deviation in parenthesis.*

| QM atom | **Enzyme** | | | | |
|---|---|---|---|---|---|
| | $Q^{AC}$ | $Q^{TS}$ | $Q^{TI}$ | $\Delta Q^{TS\text{-}AC}$ | $\Delta Q^{TI\text{-}AC}$ |
| K73-OQ1 | –0.85 (0.01) | –0.59 (0.03) | –0.52 (0.03) | 0.26 | 0.33 |
| K73-OQ2 | –0.76 (0.03) | –0.68 (0.03) | –0.67 (0.03) | 0.08 | 0.09 |
| K73-C | 0.74 (0.01) | 0.72 (0.01) | 0.71 (0.01) | –0.02 | –0.03 |
| DW-O | –0.66 (0.01) | –0.74 (0.03) | –0.58 (0.02) | –0.08 | 0.08 |
| IME-O | –0.60 (0.02) | –0.74 (0.03) | –0.87 (0.03) | –0.14 | –0.27 |
| IME-C | 0.66 (0.01) | 0.70 (0.01) | 0.74 (0.02) | 0.04 | 0.08 |
| IME-O(H) | –0.58 (0.02) | –0.60 (0.03) | –0.57 (0.02) | –0.02 | 0.01 |

*Table S7. Mulliken charges for the key QM region atoms for meropenem in the 50° orientation. DW donates a hydrogen bond to the 6α-hydroxyethyl hydroxyl group. Locations for the minima and TS: AC 0.8/3.4, TS –0.1/1.7, TI –1.0/1.5. Standard deviation in parenthesis.*

| QM atom | **Enzyme** | | | | |
|---|---|---|---|---|---|
| | $Q^{AC}$ | $Q^{TS}$ | $Q^{TI}$ | $\Delta Q^{TS\text{-}AC}$ | $\Delta Q^{TI\text{-}AC}$ |
| K73-OQ1 | –0.84 (0.03) | –0.67 (0.03) | –0.53 (-0.02) | 0.18 | 0.32 |
| K73-OQ2 | –0.76 (0.03) | –0.72 (0.03) | –0.68 (-0.03) | 0.03 | 0.07 |
| K73-C | 0.74 (0.01) | 0.72 (0.01) | 0.71 (0.01) | –0.02 | –0.03 |
| DW-O | –0.69 (0.01) | –0.62 (0.03) | –0.60 (0.02) | 0.07 | 0.09 |
| MER-O | –0.58 (0.02) | –0.78 (0.04) | –0.85 (0.04) | –0.20 | –0.27 |
| MER-C | 0.66 (0.01) | 0.72 (0.02) | 0.74 (0.02) | 0.06 | 0.08 |
| MER-O(H) | –0.51 (0.02) | –0.53 (0.02) | –0.54 (0.02) | –0.02 | –0.02 |

*Table S8. Mulliken charges for the key QM region atoms for meropenem in the 50° orientation. DW accepts a hydrogen bond from the 6α-hydroxyethyl hydroxyl group. Locations for the minima and TS: AC 0.8/3.4, TS –0.5/1.9, TI –1.0/1.5. Standard deviation in parenthesis.*

| QM atom | **Enzyme** | | | | |
|---|---|---|---|---|---|
| | $Q^{AC}$ | $Q^{TS}$ | $Q^{TI}$ | $\Delta Q^{TS–AC}$ | $\Delta Q^{TI–AC}$ |
| K73-OQ1 | –0.85 (0.03) | –0.57 (0.03) | –0.52 (0.02) | 0.28 | 0.33 |
| K73-OQ2 | –0.75 (0.03) | –0.69 (0.03) | –0.66 (0.03) | 0.07 | 0.09 |
| K73-C | 0.74 (0.01) | 0.71 (0.01) | 0.71 (0.01) | –0.03 | –0.03 |
| DW-O | –0.67 (0.02) | –0.76 (0.03) | –0.59 (0.02) | –0.08 | 0.08 |
| MER-O | –0.58 (0.03) | –0.72 (0.04) | –0.85 (0.04) | –0.14 | –0.27 |
| MER-C | 0.66 (0.01) | 0.70 (0.02) | 0.74 (0.02) | 0.04 | 0.08 |
| MER-O(H) | –0.54 (0.02) | –0.59 (0.03) | –0.58 (0.02) | –0.05 | –0.03 |

## References


1. Smith, C. A.; Stewart, N. K.; Toth, M.; Vakulenko, S. B., Structural Insights into the Mechanism of Carbapenemase Activity of the OXA-48 β-Lactamase. *Antimicrob. Agents Chemother.* **2019,** *63* (10), e01202-19.
2. Akhter, S.; Lund, B. A.; Ismael, A.; Langer, M.; Isaksson, J.; Christopeit, T.; Leiros, H. S.; Bayer, A., A Focused Fragment Library Targeting the Antibiotic Resistance Enzyme - Oxacillinase-48: Synthesis, Structural Evaluation and Inhibitor Design. *Eur. J. Med. Chem.* **2018,** *145*, 634-648.
3. Sondergaard, C. R.; Olsson, M. H.; Rostkowski, M.; Jensen, J. H., Improved Treatment of Ligands and Coupling Effects in Empirical Calculation and Rationalization of pKa Values. *J. Chem. Theory Comput.* **2011,** *7*, 2284-2295.
4. Maier, J. A.; Martinez, C.; Kasavajhala, K.; Wickstrom, L.; Hauser, K. E.; Simmerling, C., ff14SB: Improving the Accuracy of Protein Side Chain and Backbone Parameters from ff99SB. *J. Chem. Theory Comput.* **2015,** *11*, 3696-3713.
5. Vanquelef, E.; Simon, S.; Marquant, G.; Garcia, E.; Klimerak, G.; Delepine, J. C.; Cieplak, P.; Dupradeau, F. Y., R.E.D. Server: a Web Service for Deriving RESP and ESP Charges and Building Force Field Libraries for New Molecules and Molecular Fragments. *Nucleic Acids Res.* **2011,** *39*, W511-7.
6. Wang, J.; Wolf, R. M.; Caldwell, J. W.; Kollman, P. A.; Case, D. A., Development and Testing of a General Amber Force Field. *J. Comput. Chem.* **2004,** *25* (9), 1157-1174.
7. Hirvonen, V. H. A.; Mulholland, A. J.; Spencer, J.; van der Kamp, M. W., Small Changes in Hydration Determine Cephalosporinase Activity of OXA-48 β-Lactamases. *ACS Catal.* **2020,** *10* (11), 6188-6196.
8. Case, D. A.; Ben-Shalom, I. Y.; Brozell, S. R.; Cerutti, D. S.; Cheatham, T. E. I.; Cruzeiro, V. W. D.; Darden, T. A.; Duke, R. E.; Ghoreishi, D.; Giambasu, G.; Giese, T. J.; Gilson, H.; Gohlke, H.; Goetz, A. W.; Greene, D.; Harris, R.; Homeyer, N.; Huang, Y.; Izadi, S.; Kovalenko, A.; Krasny, R.; Kurtzman , T.; Lee, T. S.; LeGrand, S.; Li, P.; Lin, C.; Liu, J.; Luchko, T.; Luo, R.; Man, V.; Mermelstein, D.; Merz, K. M.; Miao, Y.; Monard, G.; Nguyen, C.; Nguyen, H.; Onufriev, A.; Pan, F.; Qi, R.; Roe, D. R.; Roitberg, A.; Sagui, C.; Schott-Verdugo, S.; Shen, J.; Simmerling, C. L.; Smith, J.; Swails, J.; Walker, R. C.; Wang, J.; Wei, H.; Wilson, L.; Wolf, R. M.; Wu, X.; Xiao, L.; Xiong, Y.; M., Y. D.; P.A., K. *AMBER 2019*, University of California: San Francisco, 2019.
9. Salomon-Ferrer, R.; Gotz, A. W.; Poole, D.; Le Grand, S.; Walker, R. C., Routine Microsecond Molecular Dynamics Simulations with AMBER on GPUs. 2. Explicit Solvent Particle Mesh Ewald. *J. Chem. Theory Comput.* **2013,** *9*, 3878-3888.
10. Salomon-Ferrer, R.; Götz, A. W.; Poole, D.; Le Grand, S.; Walker, R. C., Routine Microsecond Molecular Dynamics Simulations with AMBER on GPUs. 2. Explicit Solvent Particle Mesh Ewald. *J. Chem. Theory Comput.* **2012,** *8* (5), 1542-1555.
11. Le Grand, S.; Götz, A. W.; Walker, R. C., SPFP: Speed Without Compromise—A Mixed Precision Model for GPU Accelerated Molecular Dynamics Simulations. *Comput. Phys. Commun.* **2013,** *184*, 374-380.
12. Roe, D. R.; Cheatham, T. E., PTRAJ and CPPTRAJ: Software for Processing and Analysis of Molecular Dynamics Trajectory Data. *J. Chem. Theory Comput.* **2013,** *9* (7), 3084-3095.
13. Elstner, M.; Porezag, D.; Jungnickel, G.; Elsner, M.; Haugk, M.; Frauenheim, T. H.; Suhai, S.; Seifert, G., Self-Consistent-Charge Density-Functional Tight-Binding Method for Simulations of Complex Materials Properties. *Phys. Rev. B* **1998,** *58* (11), 7260-7268.
14. Elstner, M.; Frauenheim, T. H.; Kaxiras, E.; Seifert, G.; Suhai, S., A Self-Consistent Charge Density-Functional Based Tight-Binding Scheme for Large Biomolecules. *Phys. Status Solidi* **2000,** *217*, 357-376.
15. Grossfield, A. WHAM: an Implementation of the Weighted Histogram Analysis Method. http://membrane.urmc.rochester.edu/content/wham/ (accessed 08/01/2020).
16. Marcos-Alcalde, I.; Setoain, J.; Mendieta-Moreno, J. I.; Mendieta, J.; Gomez-Puertas, P., MEPSA: Minimum Energy Pathway Analysis for Energy Landscapes. *Bioinformatics* **2015,** *31*, 3853-3855.

17. Hirvonen, V. H. A.; Hammond, K.; Chudyk, E. I.; Limb, M. A. L.; Spencer, J.; Mulholland, A. J.; van der Kamp, M. W., An Efficient Computational Assay for β-Lactam Antibiotic Breakdown by Class A β-Lactamases. *J. Chem. Inf. Model.* **2019,** *59* (8), 3365-3369.
18. Oueslati, S.; Nordmann, P.; Poirel, L., Heterogeneous Hydrolytic Features for OXA-48-like β-lactamases. *J. Antimicrob. Chemother.* **2015,** *70* (4), 1059-1063.
19. Frisch, M. J.; Trucks, G. W.; Schlegel, H. B.; Scuseria, G. E.; Robb, M. A.; Cheeseman, J. R.; Scalmani, G.; Barone, V.; Petersson, G. A.; Nakatsuji, H.; Li, X.; Caricato, M.; Marenich, A. V.; Bloino, J.; Janesko, B. G.; Gomperts, R.; Mennucci, B.; Hratchian, H. P.; Ortiz, J. V.; Izmaylov, A. F.; Sonnenberg, J. L.; Williams-Young, D.; Ding, F.; Lipparini, F.; Egidi, F.; Goings, J.; Peng, B.; Petrone, A.; Henderson, T.; Ranasinghe, D.; Zakrzewski, V. G.; Gao, J.; Rega, N.; Zheng, G.; Liang, W.; Hada, M.; Ehara, M.; Toyota, K.; Fukuda, R.; Hasegawa, J.; Ishida, M.; Nakajima, T.; Honda, Y.; Kitao, O.; Nakai, H.; Vreven, T.; Throssell, K.; Montgomery, J. A. J.; Peralta, J. E.; Ogliaro, F.; Bearpark, M. J.; Heyd, J. J.; Brothers, E. N.; Kudin, K. N.; Staroverov, V. N.; Keith, T. A.; Kobayashi, R.; Normand, J.; Raghavachari, K.; Rendell, A. P.; Burant, J. C.; Iyengar, S. S.; Tomasi, J.; Cossi, M.; Millam, J. M.; Klene, M.; Adamo, C.; Cammi, R.; Ochterski, J. W.; Martin, R. L.; Morokuma, K.; Farkas, O.; Foresman, J. B.; Fox, D. J. *Gaussian 16, Revision A.03*, Gaussian Inc.: Wallingford CT, 2016.
20. Gerenkamp, M.; Grimme, S., Spin-component Scaled Second-Order Møller–Plesset Perturbation Theory for the Calculation of Molecular Geometries and Harmonic Vibrational Frequencies. *Chem. Phys. Lett.* **2004,** *392*, 229-235.
21. Dunning, T. H., Gaussian basis sets for use in correlated molecular calculations. I. The atoms boron through neon and hydrogen. *J. Chem. Phys.* **1989,** *90* (2), 1007-1023.
22. Neese, F., The ORCA Program System. *WIREs Comput. Mol. Sci.* **2012,** *2*, 73-78.
23. Neese, F., Software Update: the ORCA Program System, Version 4.0. *WIREs Comput. Mol. Sci.* **2017,** *8*, e1327.
24. Zhao, Y.; Truhlar, D. G., The M06 suite of density functionals for main group thermochemistry, thermochemical kinetics, noncovalent interactions, excited states, and transition elements: two new functionals and systematic testing of four M06-class functionals and 12 other functionals. *Theor. Chem. Acc.* **2007,** *120* (1-3), 215-241.
25. Weigend, F.; Furche, F.; Ahlrichs, R., Gaussian Basis Sets of Quadruple Zeta Valence Quality for Atoms H–Kr. *J. Chem. Phys.* **2003,** *119*, 12753-12762.
